# Altered microRNA and mRNA profiles during heart failure in the human sinoatrial node

**DOI:** 10.1038/s41598-021-98580-x

**Published:** 2021-09-29

**Authors:** Ning Li, Esthela Artiga, Anuradha Kalyanasundaram, Brian J. Hansen, Amy Webb, Maciej Pietrzak, Brandon Biesiadecki, Bryan Whitson, Nahush A. Mokadam, Paul M. L. Janssen, John D. Hummel, Peter J. Mohler, Halina Dobrzynski, Vadim V. Fedorov

**Affiliations:** 1 Department of Physiology and Cell Biology, The Ohio State University College of Medicine and Wexner Medical Center, Columbus, OH 43210-1218 USA; 2 Bob and Corrine Frick Center for Heart Failure and Arrhythmia, Dorothy M. Davis Heart & Lung Research Institute, The Ohio State University College of Medicine and Wexner Medical Center, Columbus, OH USA; 3 Biomedical Informatics Shared Resources, The Ohio State University College of Medicine and Wexner Medical Center, Columbus, OH USA; 4 Department of Surgery, Division of Cardiac Surgery, The Ohio State University College of Medicine and Wexner Medical Center, Columbus, OH USA; 5 Department of Internal Medicine, Division of Cardiovascular Medicine, The Ohio State University College of Medicine and Wexner Medical Center, Columbus, OH USA; 6 Division of Cardiovascular Sciences, University of Manchester, Manchester, UK; 7 Department of Anatomy, Jagiellonian University Medical College, Cracow, Poland

**Keywords:** Translational research, Bioinformatics, Molecular medicine, Cardiovascular diseases

## Abstract

Heart failure (HF) is frequently accompanied with the sinoatrial node (SAN) dysfunction, which causes tachy-brady arrhythmias and increased mortality. MicroRNA (miR) alterations are associated with HF progression. However, the transcriptome of HF human SAN, and its role in HF-associated remodeling of ion channels, transporters, and receptors responsible for SAN automaticity and conduction impairments is unknown. We conducted comprehensive high-throughput transcriptomic analysis of pure human SAN primary pacemaker tissue and neighboring right atrial tissue from human transplanted HF hearts (n = 10) and non-failing (nHF) donor hearts (n = 9), using next-generation sequencing. Overall, 47 miRs and 832 mRNAs related to multiple signaling pathways, including cardiac diseases, tachy-brady arrhythmias and fibrosis, were significantly altered in HF SAN. Of the altered miRs, 27 are predicted to regulate mRNAs of major ion channels and neurotransmitter receptors which are involved in SAN automaticity (e.g. HCN1, HCN4, SLC8A1) and intranodal conduction (e.g. SCN5A, SCN8A) or both (e.g. KCNJ3, KCNJ5). Luciferase reporter assays were used to validate interactions of miRs with predicted mRNA targets. In conclusion, our study provides a profile of altered miRs in HF human SAN, and a novel transcriptome blueprint to identify molecular targets for SAN dysfunction and arrhythmia treatments in HF.

## Introduction

Heart failure (HF) is one of the leading causes of morbidity and mortality in the United States and globally, which continues to increase as the population ages^1^. Patients with HF frequently have abnormalities of the pacemaker and conduction system, which make them more vulnerable to fatal arrhythmias. In fact, ~ 20–50% of sudden cardiac deaths result from brady-arrhythmias that are commonly related to sinoatrial node (SAN) dysfunction^2–5^. Treatments like defibrillators and cardiac resynchronization therapy significantly reduce total mortality and hospitalizations in advanced HF patients^2^. This evidence suggests that maintenance of normal heart rhythm and conduction may be crucial to improve/preserve cardiac function in HF patients. As such, understanding the mechanism of HF induced SAN impairment and related conduction system disorders, could provide novel strategies for anti-arrhythmic treatments in HF. However, there is a paucity of information regarding the physiologic and pathophysiologic features of the human SAN pacemaker-conduction complex at the molecular, cellular, and tissue levels^6^.

Intrinsic mechanisms of SAN impairments in HF, such as fibrosis and molecular remodeling, have been the topic of multiple studies in animal models^7^. However, the large interspecies variation hinders the integration and translation of these animal model findings to their clinical application^8^. Recent animal studies reported that microRNAs (miRs) play an important role in exercise training induced bradycardia^9^ or SAN dysfunction^10–12^ through the regulation of important ion channels like HCN4, Cav1.2 and Cav1.3. Furthermore, several human tissue studies have shown miRs to be differentially expressed in the isolated SAN and atrial fibroblasts^13^, atria and ventricle tissue during HF^14–16^, suggesting the possible involvement of miRs in the pathogenesis of HF, and as potential biomarkers and therapeutic targets. However, transcriptional profiles of myocardial mRNAs and miRs in human HF SAN are unknown.

Our recent studies of ex-vivo human hearts^17–20^ provided an unprecedented opportunity to apply high-resolution near-infrared optical mapping, 3D structural imaging, and molecular mapping to resolve functional and molecular mechanisms of HF-associated remodeling across intranodal pacemakers and conduction pathways of the human SAN complex^17^. These studies revealed that in normal conditions, the leading pacemaker cluster is located in the central compartment of the human SAN complex, which has the highest expression of all 3 cardiac HCN isoforms (HCN1, HCN2, and HCN4) than other SAN compartments (e.g. SAN head, tail or sinoatrial conduction pathways) and surrounding atria^18^. Furthermore, heterogeneous expression of neurotransmitter receptors and associated ion channels (e.g. adenosine A1 receptor and downstream GIRK4 channel protein) across the SAN complex could allow intranodal shifts of the leading pacemaker and preferential conduction pathways, which play important roles in robust physiological SAN rhythm regulation and/or protection during diseased conditions^17^. Recently, we found that both neuronal (Nav1.6) and cardiac (Nav1.5) voltage gated sodium channels are essential for preventing failure of conduction and pacemaking in the human SAN complex during physiological and pathophysiological perturbations^19^. Specifically, neuronal Nav1.6 (encoded by SCN8A) is downregulated in HF SAN, which is associated with intranodal conduction impairment.

Although these previous studies revealed heterogeneous expressions of many proteins across the human SAN complex and their roles in pacemaking and conduction, the underlying causes of these protein level changes, in association to miR alterations, are still unknown.

The objective of the present study is to identify differences in the transcriptomic profile (mRNA and miR) of human HF vs non-failing (nHF) SANs, which can be used to identify potential SAN-specific molecular targets for modifying SAN function in HF patients. HF and nHF RA tissues neighboring the SAN were also included to define unique SAN transcriptomic profiles and tissue-specific remodeling in HF. Importantly, RA-specific molecular profile differences between HF and nHF hearts can be used to identify SAN-specific miRs to modulate mRNAs and their expression only in the SAN without affecting the surrounding RA.

In the present study, the miR and mRNA profiles were analyzed with purified samples from human SAN central compartment of HF transplant hearts and donor hearts without HF and arrhythmia history. Our analyses revealed the unique transcriptomic profile of human SAN and SAN-specific altered miRs in HF, of which, those targeting ion channels and receptors may be responsible for the impairment of SAN automaticity and conduction, and further confirmed with Luciferase assays. Accumulatively, our results provide a transcriptome blueprint to identify novel molecular targets for SAN dysfunction treatments and new insights into the mechanisms involved in HF remodeling of the SAN pacemaker-conduction complex.

## Results

### Extraction of pure SAN pacemaker tissue from the central compartment of the human SAN complex

**Figure 1:**
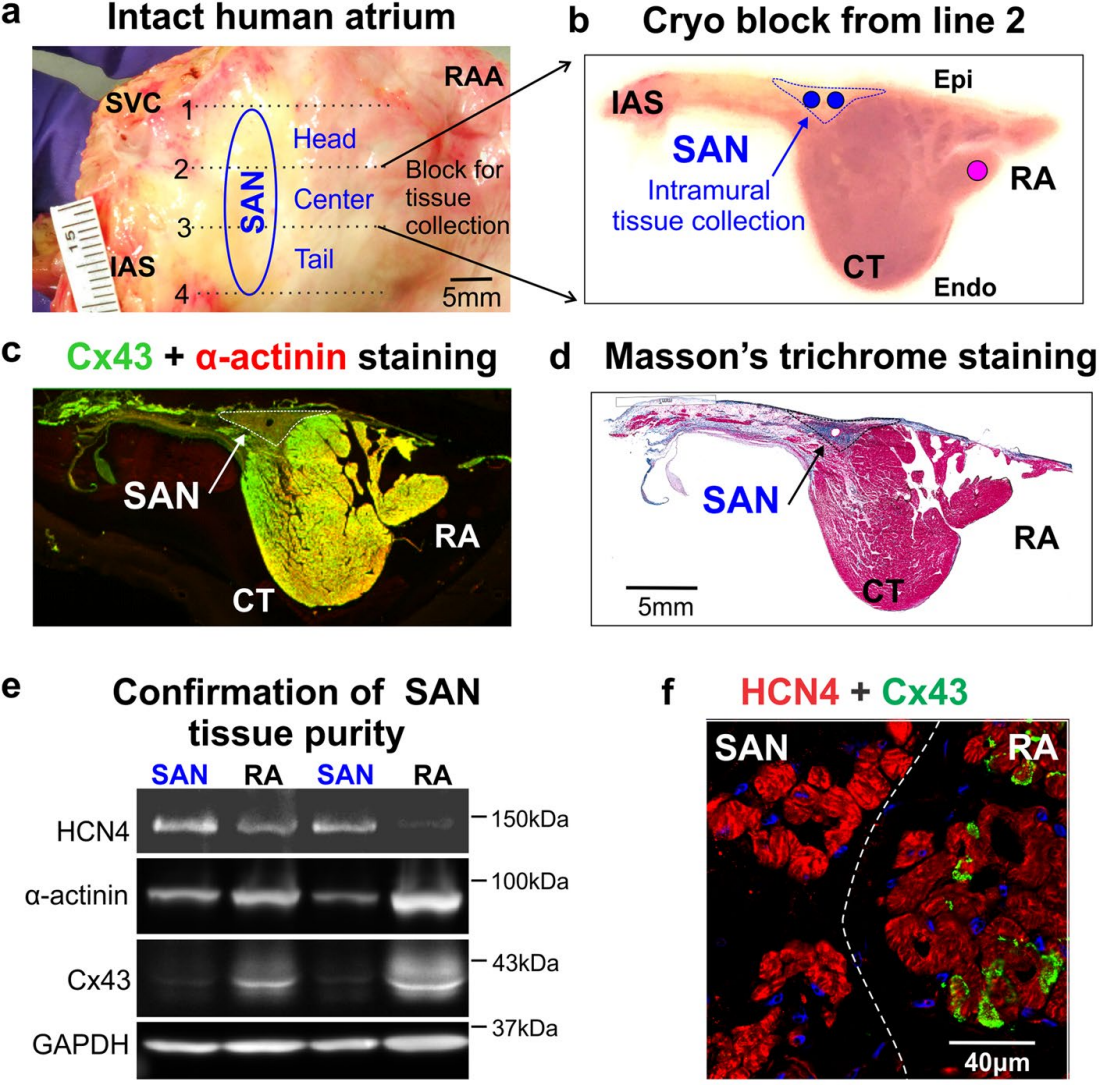
Human SAN pacemaker tissue extraction and validation. (**a**) Image of a human right atrial (RA) preparation. The location of the SAN is represented by the blue oval. The whole SAN pacemaker complex was embedded in a freezing medium (OCT) and cut into three cryo-blocks (head, center and tail). (**b**) Cross view of the SAN center cryo-block cut from line 2 as shown in panel A; blue dotted line indicates the SAN area and the blue and pink circles indicate the locations for tissue biopsy of the SAN and RA, respectively. (**c**) Connexin-43 (Cx43, green) and α-actinin (red) immunostaining show the SAN as a Cx43 negative and α-actinin positive region. (**d**) Masson’s trichrome staining shows the SAN as a highly fibrotic region compared to the atria. (**e**) Representative immunoblots of the SAN versus RA samples with the anti-bodies of HCN4, α-actinin, connexin-43, and GAPDH. The uncropped full-length blots are presented in Supplemental Information file Fig. S1. (**f**) Immunostaining with HCN4 (red) and Cx43 (green) shows negative Cx43 and higher HCN4 expression in the SAN than the RA. CT indicates crista terminalis; Endo/Epi, endo/epicardial; GAPDH, glyceraldehyde 3-phosphate dehydrogenase; HCN4, hyperpolarization-activated cyclic nucleotide-gated channel 4; HF, heart failure; IAS, interatrial septum; RA (A), right atrial (appendage); SAN, sinoatrial node; SVC, superior vena cava.

The first step in our goal to identify SAN-specific miRs was to extract pure SAN pacemaker tissue from the central SAN compartment without contamination from surrounding RA tissues in both HF (n = 10) and nHF (n = 9) hearts (see Table S1 for clinical information). We chose to use the central SAN compartment since our recent molecular and functional mapping studies of ex-vivo human SAN^17^, show that the leading SAN pacemaker is primarily located in the central intramural pacemaker compartment. As shown in Fig. 1 cryo-sections were collected from both ends of the cryo-block containing the central intranodal pacemaker, which was defined as a Cx43-negative but α-actinin positive region by immunolabeling (Fig. 1c), and as a compact fibrotic region by Masson’s trichrome staining (Fig. 1d). Furthermore, since protein and total RNA were isolated from the same SAN sample, we also used immunoblotting to verify that collected SAN tissue was free from atrial myocardial tissue contamination, as described previously^18^. Immunoblotting confirmed that all HF and nHF SAN samples (n = 19) have significantly lower Cx43 expression relative to paired RA, while both SAN and atria tissue samples showed comparable α-actinin expression (Fig. 1e). Full-length blots are presented in Supplemental Information file Fig. S1. In all 19 hearts, total Cx43 expression was significantly lower in the SAN tissue vs the RA, with a GAPDH normalized band density ratio of SAN/RA = 0.33 ± 0.04 (n = 19, p < 0.001), which demonstrates the purity of the samples. In contrast, HCN4 expression by immunoblotting and immunostaining was significantly higher in the SAN center than in the RA, with a GAPDH normalized band density ratio of SAN/RA = 2.7 ± 1.7 (n = 19, p < 0.001) (Fig. 1e,f).

### Differential profiles of mRNA and miR in human SAN versus RA tissue

**Figure 2:**
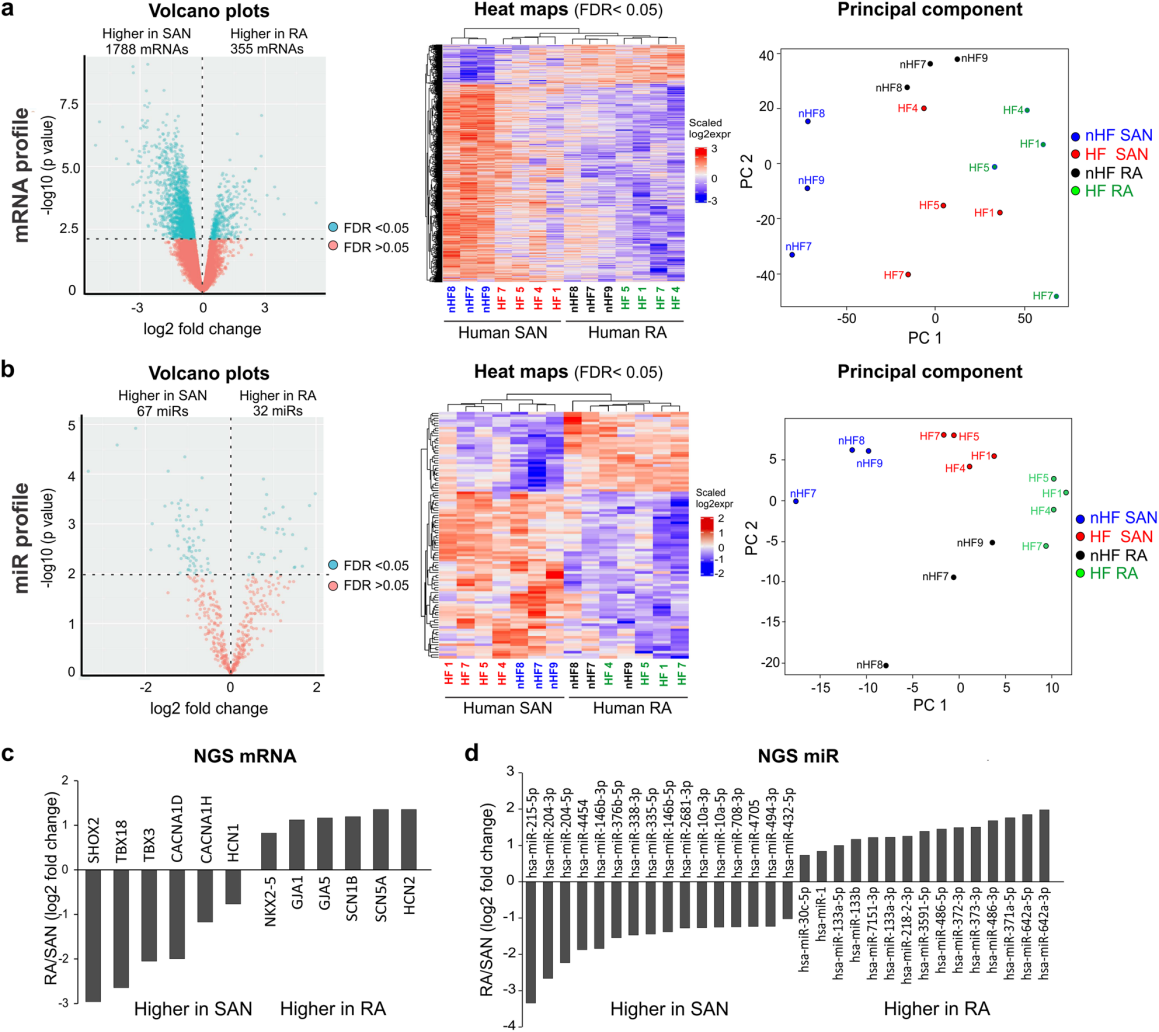
Comparison of miR and mRNA profiles of the human SAN vs RA. (**a**) Volcano plot (left) of mRNA profile for paired human SAN and RA (n = 7). Heat map (middle) of significantly different mRNAs for paired SAN and RA. PCA (right) for SAN and RA mRNA samples; each circle represents a single sample. (**b**) Volcano plot (left) of miR profile for paired human SAN and RA. Heat map (middle) of significantly different miRs for paired SAN and RA. PCA (right) for SAN and RA miR samples; each circle represents an individual sample. (**c**) Bar graphs show representative significantly different (FDR < 0.05) mRNAs and (**d**) miRs in the human SAN vs. RA detected by NGS. Plots and heat maps were generated with R 3.6.3. HF indicates heart failure; NGS, next generation sequencing; PCA, principal component analysis; qPCR, quantitative polymerase chain reaction; RA, right atrium; and SAN, sinoatrial node.

Next, in order to define the distinct expression profiles of mRNA and miRs in both HF and nHF SAN and neighboring RA tissue, the collected SAN (n = 7) and RA samples (n = 7) were studied using next generation sequencing (NGS). Since the accuracy of NGS depends on the quality of RNA samples, only the SAN samples with RNA integrity number (RIN) higher than 5.8 and their paired RA samples were used for NGS analysis. Furthermore, donors with history of alcohol use patients^19^ and cardiac disease were also excluded from NGS analysis. At the transcriptional level, > 15,000 genes per sample were identified and compared between human SAN and RA tissue. Our results revealed that 1788 mRNAs were significantly more abundant (false discovery rate (FDR) < 0.05) in the SAN than the paired atrial sample (n = 7). On the other hand, 355 mRNAs were significantly less abundant (FDR < 0.05) in the SAN compared to its own atrial sample (Fig. 2a, Online Table 1). NGS detected that genes important for SAN development and function such as HCN1, CACNA1D (Cav1.3), TBX3, TBX18, and Shox2^6,21^, had higher transcriptional levels in the human SAN, while mRNAs important for atrial development and function such as NKX2-5, GJA1 (Cx43), GJA5 (Cx40), SCN5A and SCN1B were significantly higher in the human atria (Fig. 2c). PCR validation of the SAN-specific key mRNAs is shown in Fig.S2.

Out of > 2500 human miRs examined, 67 were significantly more abundant and 32 significantly less abundant (FDR < 0.05) in the SAN (n = 7) than the paired atrial sample (Fig. 2b; Online Table 1). Principal component analysis (PCA) showed clear clusters of distinguished SAN or RA samples. Figure 2d shows representative differently expressed miRs in the human SAN vs RA. Core Analysis of altered miR targets (FDR < 0.05) by Ingenuity Pathway Analysis (IPA) software showed that up-stream regulators include miR-1-3p, miR-30c-5p, miR-338-3p and let-7c-5p. Overall, these results suggest that the human SAN could have a unique transcriptomic profile compared with that of the surrounding RA tissue.

### HF associated alterations of mRNA and miR in the human SAN

**Figure 3:**
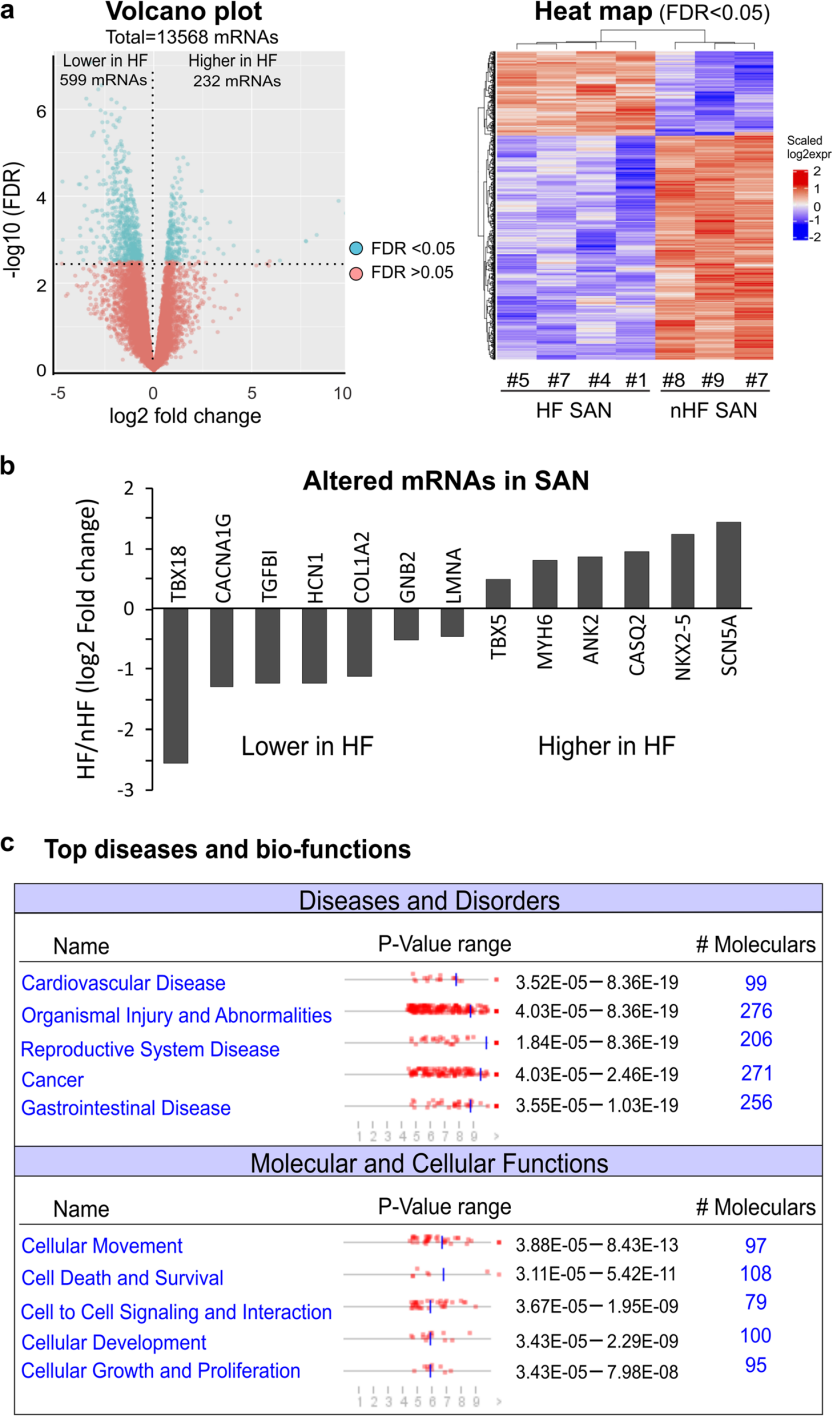
Altered mRNAs and related pathways in the human HF SAN. (**a**) Volcano plot (left) and heat map (right) of altered mRNAs in the HF human SAN; there are 599 mRNAs significantly downregulated and 232 mRNAs upregulated in the HF human SAN. (**b**) Bar graph shows NGS data of some significantly altered genes related to SAN dysfunction. (**c**) IPA shows the most common diseases and disorders and the top molecular and cellular functions that are related to the significantly altered mRNAs in the HF human SAN. Plots and heat maps were generated with R 3.6.3.

**Figure 4:**
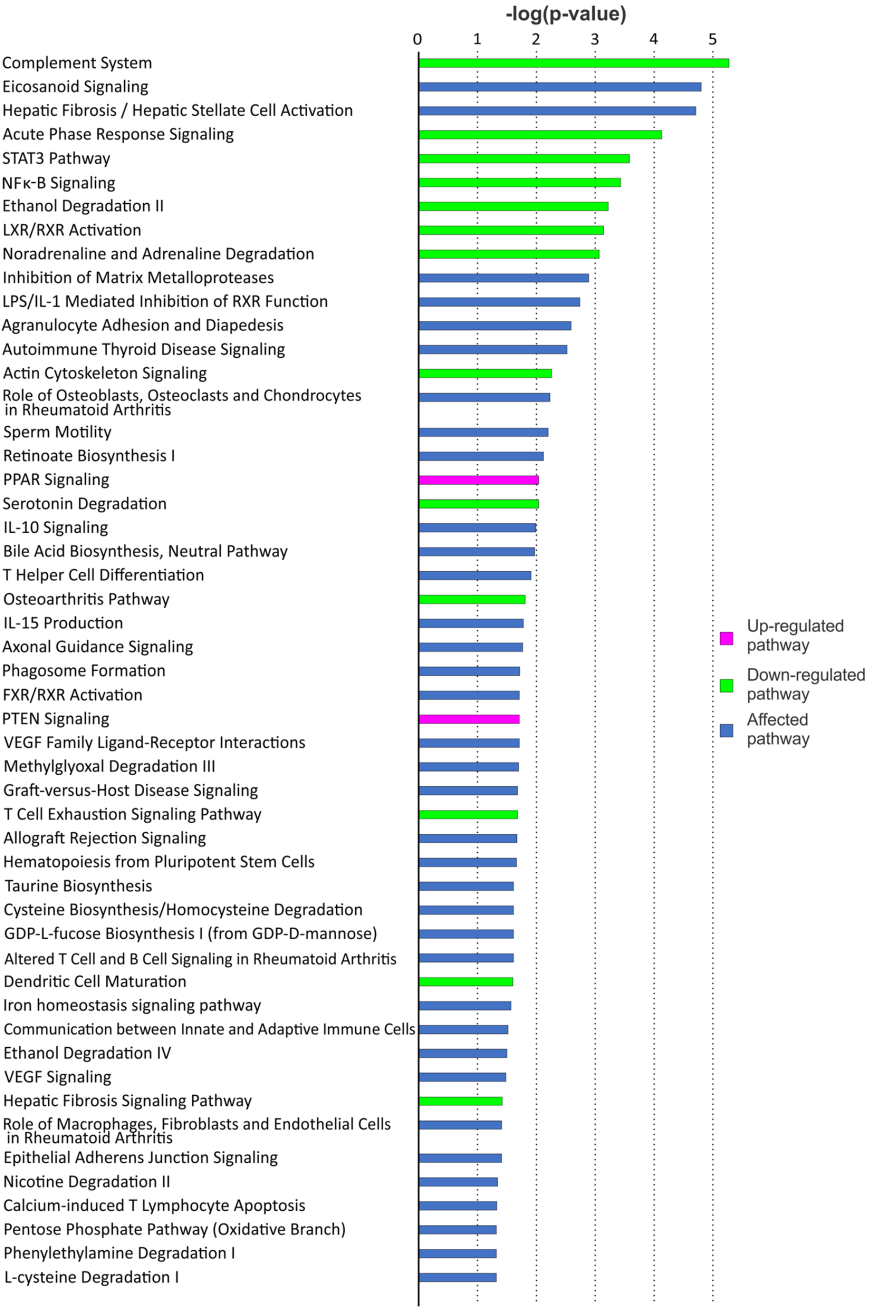
Signaling pathways in HF human SAN predicted by IPA. HF indicates heart failure; IPA, Ingenuity pathway analysis; SAN, sinoatrial node. The graph was generated with IPA (Winter Release December 2019, Qiagen).

We next investigated the alterations in the SAN and RA transcriptomic profiles specifically associated with HF. When comparing mRNA profiles of HF (n = 4) vs nHF (n = 3) SAN, 832 altered mRNAs in the SAN (FDR < 0.05) were associated with HF, as demonstrated in Fig. 3 and Online Table 1. As shown in Fig. 3b, some of the genes that are important for maintaining normal SAN rhythm and cardiac conduction system function^6,21–24^ are altered in HF. IPA with 290 mRNAs having log2 fold changes greater than 1.5, showed multiple dysregulated signaling pathways in HF SAN (Fig. 4). Multiple altered pathways were related to inflammation and immune response signaling. Moreover, the top three diseases and bio-function pathways identified were: 1) cardiovascular disease, 2) organismal injury and abnormalities, and 3) reproductive system disease (Fig. 3c). Necrosis was also predicted to be increased (z-score 2.318), and 52 out of 98 genes had measurement direction consistent with an increase in necrosis (overlap p-value 5.42E-11).

**Figure 5:**
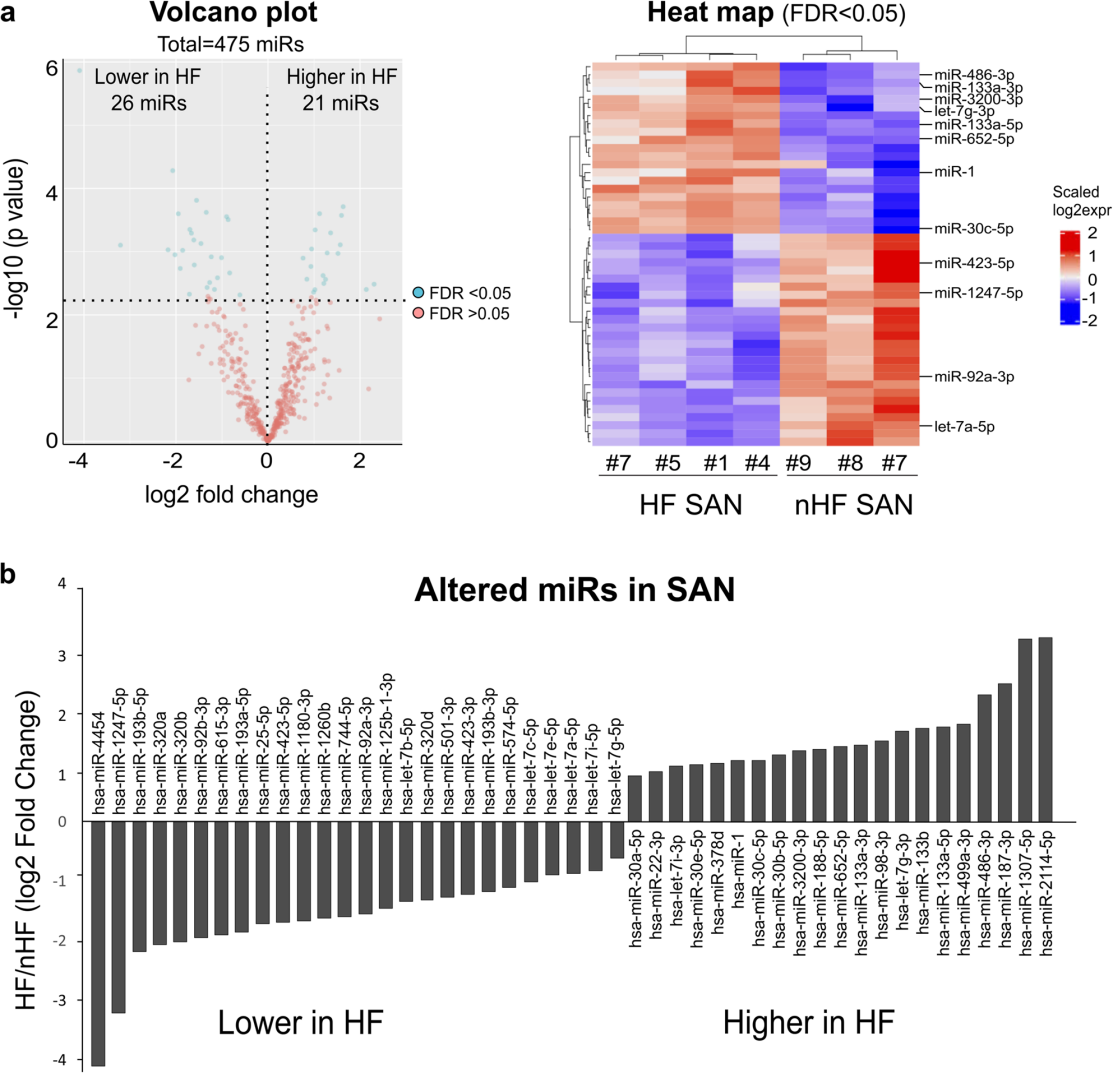
Altered miRs in HF human SAN. (**a**) Volcano plot (left) and heat map (right) of altered miRs in the HF human SAN. (**b**) Bar graph shows significantly altered miRs during HF in the human SAN as detected by NGS. Plots and heat maps were generated with R 3.6.3. HF indicates heart failure; NGS, next generation sequencing; SAN, sinoatrial node.

**Figure 6:**
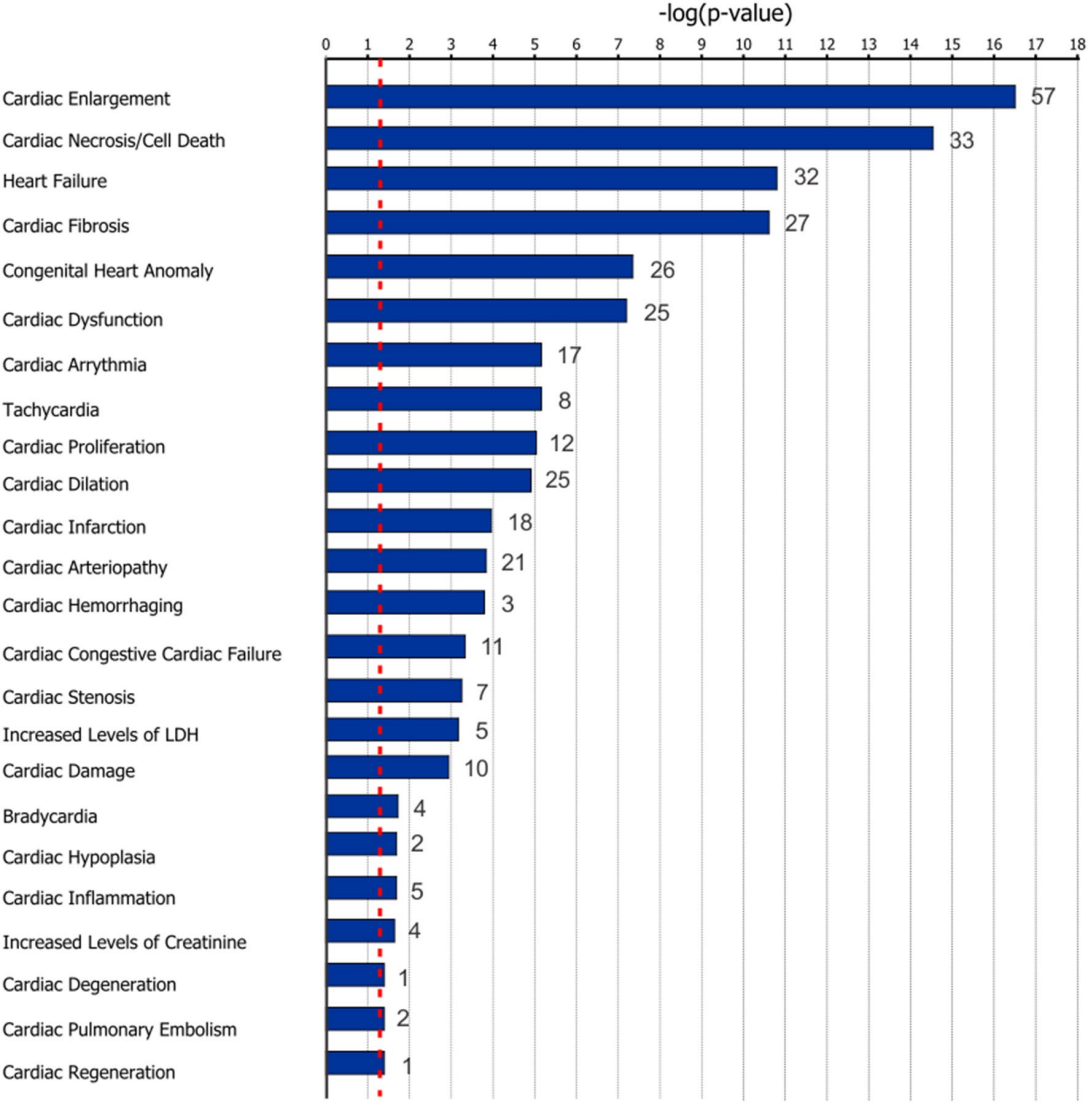
Cardiac diseases related to mRNAs predicted to be targets of altered miRs in HF human SAN. Numbers after each bar indicate the total IPA-predicted mRNA targets of significantly altered miRs (P < 0.05) involved in each disease. The graph was generated with IPA (Winter Release December 2019, Qiagen). HF indicates heart failure; LDH, lactate dehydrogenase; SAN, sinoatrial node.

For NGS miRs data, there was a downregulation of 26 miRs and an upregulation of 21 miRs in the human SAN associated with HF (Fig. 5a, b; Online Table 1). Targeting information was available for 35 out of 47 of these altered miRs in the IPA database (18 downregulated and 17 upregulated), which predicted that these 35 miRs may target > 12,000 mRNAs. Among significantly changed mRNAs detected by NGS, 564 mRNAs were predicted as targets of these 35 significantly altered miRs. Experimentally, 38 mRNAs have been found to be regulated by 7 miRs in the IPA database (Online Table 2). To assess the potential global signaling network and alterations in response to the significantly changed miRs, all experimentally confirmed miR targets (465 mRNA) in IPA database were utilized for pathway analysis. The results showed mir-1-3p, let-7a-5p, mir-30c-5p, and mir133-3p and their predicted targets were mainly involved in developmental disorders and organismal injury and abnormalities. Figure 6 shows multiple cardiac diseases related to predicted mRNAs targets of the significantly altered miRs in human HF SAN. Cardiac dilation, necrosis, fibrosis and tachy-brady arrhythmias signaling pathways were predicted as top canonical pathways (p < 0.001).

**Figure 7:**
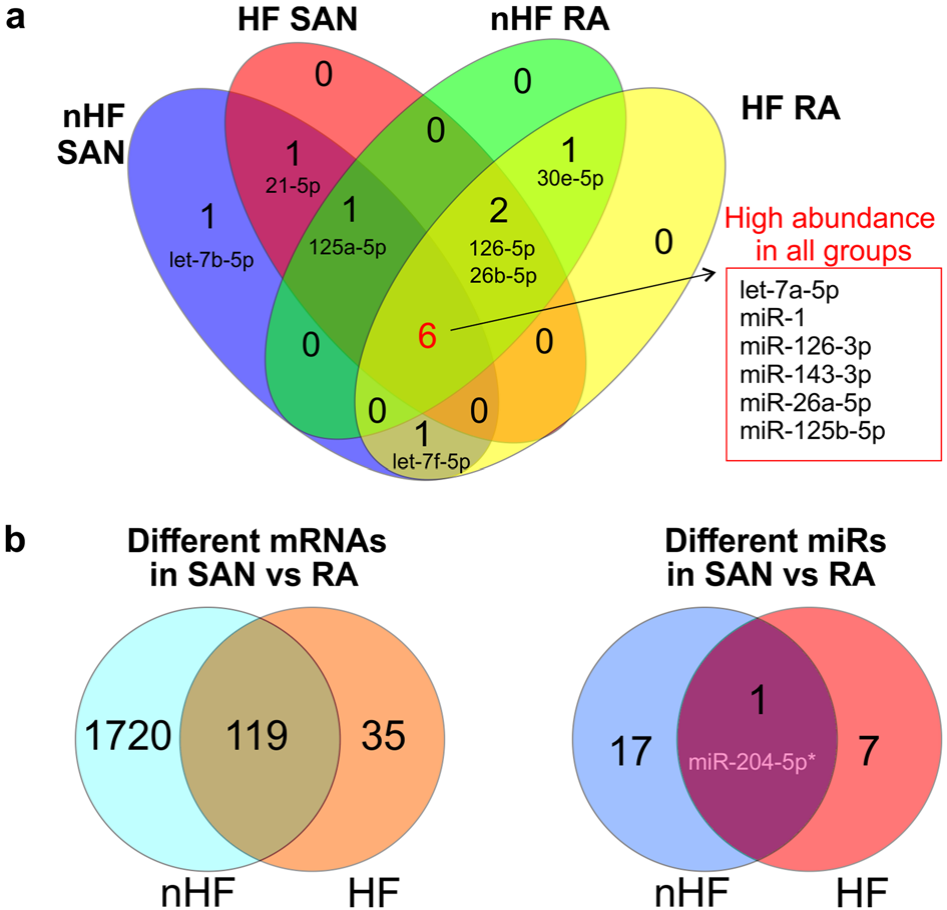
Similarity of miR and mRNA profiles in SAN vs RA in HF and nHF hearts. (**a**) Top 10 most abundant miRs revealed by NGS in each HF-RA, nHF-RA, HF-SAN, and nHF-SAN groups. (**b**) Comparison of significantly different mRNAs (left) and miRs (right) in the SAN vs RA between control and HF groups. HF indicates heart failure; NGS, next generation sequencing; RA, right atrium; SAN, sinoatrial node.

Top 10 (Fig. 7a) and top 50 (Online Table 3) of the most abundant miRs in each group were compared between all four groups. Although some miRs, such as let-7a-5p and miR-1, are significantly altered in HF SAN vs nHF, they are still highly abundant in all four groups. In nHF hearts, there were 18 significantly different miRs in SAN vs paired RA, but in HF hearts only 8 miRs were different in SAN and paired RA. Moreover, only miR-204-5p was higher in the SAN vs the RA in both the nHF and HF groups as detected by NGS. qPCR also confirmed significantly higher expression of miR-204-5p in the human SAN than matched RA. At transcriptional level, HF also diminished the differences between SAN and matched RA with 1839 vs 154 different mRNAs in each comparison (Fig. 7b).

### Alteration of miRs in HF SAN associated with known targets of SAN dysfunction

**Figure 8:**
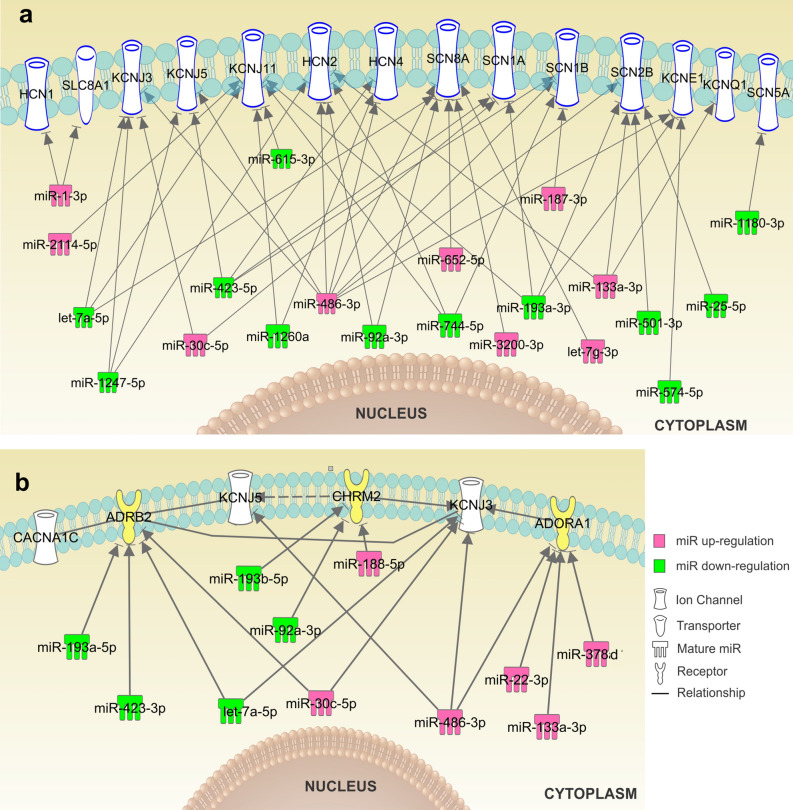
Altered miRs in HF and their targets involved in SAN automaticity and conduction. (**a**) Significantly downregulated (green) and upregulated (pink) miRs in the human HF vs nHF SAN detected by NGS. The mRNA targets of these miRs include the major ion channels involved in pacemaking and conduction of the human SAN predicted by IPA. (**b**) IPA predicted neurotransmitter receptors (and ion channels regulated by those receptors) targeted by miRs significantly altered in the HF SAN. Graphs were generated with IPA (Winter Release December 2019, Qiagen). HF indicates heart failure; IPA, Ingenuity pathway analysis; NGS, next generation sequencing; SAN, sinoatrial node.

Next, we determined if the HF associated alterations in miR expression can be correlated with known molecular targets of SAN dysfunctions. Ion channels (e.g. funny current channels HCN4, Ca^2+^-handling protein, and K^+^ channels) and neurotransmitter receptors responsible for SAN automaticity and conduction are highly enriched in the SAN compared to the RA, and alterations in their expression or function can lead to severe functional impairments^6^. In the HF SAN, 35 significantly altered miRs were predicted to target 246 mRNAs of ion channels (Online Table 4) by IPA analysis. At least 27 of the altered miRs (Online Table 5) were predicted to directly regulate mRNAs of cell surface ion channels (e.g. HCN1, HCN4, SCN5A, SCN1A, SCN8A, KCNJ3, KCNJ5) and neurotransmitter receptors (e.g. ADRB2, CHRM2, and ADORA1) involved in SAN automaticity and conduction based on previous studies^6,17,19,25^ and mRNA 3′UTR target sites (Fig. 8). With the exception of SLC8A1 (encodes Na^+^-Ca^2+^ exchange channel), IPA analysis did not discover other cell surface Ca^2+^ channel genes (e.g. CACNA1C, CACNA1D, CACNA1G) as direct targets of the significant altered miRs. Significant upregulation of miR-3200-3p, let-7g-3p, miR-486-3p, miR-652-5p, miR-133a-3p, miR-1-3p, mir-30c-5p, and miR-187-3p in the HF SAN was predicted to downregulate expression of the ion channels HCN1/4(I_f_ ), and SCN1A, SCN8A, SCN1B, SCN2B (I_Na_), which are important for cardiomyocyte pacemaking and conduction. Meanwhile, let-7a-5p, miR-1247-3p, miR-423-5p, mir-574-5p, and mir-25-5p, which are predicted to target KCNJ3/KCNJ5 genes (coding GIRK1/GIRK4), and KCNQ1/KCNE1 genes (coding IKs), were significantly downregulated in HF. These potassium channels could significantly affect the repolarization of the cardiomyocytes and affect SAN automaticity. **Fig. S3** shows PCR validation of several significantly altered miRs.

### Luciferase reporter assay to confirm predicted interactions between miRs and their target mRNAs

To investigate whether miRs of interest selected from NGS analyses can affect predicted mRNAs, Luciferase reporter assays were used to test interactions: 1) miR-3200-3p, miR-486-3p, miR-652-5p, and let-7g-3p, with their predicted target SCN8A; 2) miR-486-3p and miR-1247-5p with their predicted target KCNJ5. SCN8A and KCNJ5 encode the voltage-gated sodium channel Nav1.6 and G protein-activated inward rectifier potassium channel GIRK4 respectively. Our previous study^17,19^ found that both channels are critically important in maintaining the robustness and intranodal conduction of the human SAN. Our recent study also showed that transcription of SCN8A was significantly downregulated in the SAN of HF patients^19^.

Figure S4-S5 showed the predicted binding sites for the miRs of interest with respect to the 3′UTR of SCN8A and KCNJ5. As shown in Fig. S4b, miR-3200-5p, miR-486-3p and let-7g-3p mimics significantly repressed SCN8A 3′UTR-Luciferase expression compared to scramble miR, of which let-7g-3p showed largest effect (p < 0.01). miR-652-5p mimics did not repress SCN8A 3′UTR-Luciferase expression compared to scramble miR. Similarly, miR-1247-5p mimic could significantly repress KCNJ5 3′UTR-Luciferase expression (Fig. S5) and confirmed the IPA predicted mRNA target (Fig. 8).

## Discussion

For the first time, we revealed the transcriptional alterations of miR and mRNA profiles in the human SAN during HF, as well as altered signaling pathways associated with multiple cardiac diseases by NGS and IPA. Importantly, we revealed HF-associated miR alterations in primary SAN intranodal pacemaker tissue, which may be involved in remodeling of ion channels and receptors responsible for SAN automaticity and conduction. Our findings present a transcriptomic blueprint for identifying novel molecular targets for tachy-brady arrhythmia treatments, and provide new insights into the mechanisms involved in SAN automaticity and conduction remodeling during HF, which was absent in previous studies of HF in humans.

Chronic HF is a progressive disease that increases morbidity and early mortality. For instance, the COMPANION trial showed that the one-year mortality rate of New York Heart Association (NYHA) functional class III-IV HF patients is 19% even with optimal pharmacological therapy^2^. Of these fatalities, approximately one-half to two-thirds can be classified as sudden deaths caused by fatal arrhythmias^3^. Luu et al.^4^ reported that in advanced HF patients, 43% of the initial rhythm at the time of cardiac arrest was sinus bradycardia, suggesting impaired function of the SAN. Moreover, Sanders et al^5^ demonstrated a reduction of SAN reserve in patients with congestive HF, including prolongation of the intrinsic SAN cycle length, SAN recovery time, SAN conduction time, and abnormal propagation of the SAN impulse. Although fibrosis and ion channel remodeling were suggested to play a role in SAN dysfunction (SND) progression in HF^7^, the regulation of up-stream miRs in the mechanism of SND in HF patients is unknown.

MiRs have been implicated in cardiac development, cardiac arrhythmias, myocardial infarction, and HF. Previous miR microarray profiling and qPCR array studies have examined the potential utility of miRs as biomarkers from various HF tissue samples^14–16^. Most of the studies used blood samples (serum, plasma, or blood mononuclear cell) or ventricular biopsies, which reported clusters of miRs that could distinguish HF from nHF subjects or that could provide further differentiation between HF with preserved ejection fraction (HFpEF) and reduced ejection fraction (HFrEF). Many of these miRs appear to target important genes that are involved in cardiac hypertrophy, apoptosis, and remodeling. For example, miR-1, miR-30a, and miR-92a target genes involved in cardiac hypertrophy and apoptosis signaling^26–28^.

In the present study, by using NGS analysis, we revealed distinct miR and mRNA profiles between the human SAN vs RA in HF and nHF hearts, and HF associated miR and mRNA alterations in the human SAN. We found that miR-1 and miR-133 (known as muscle-specific myomiRs^15^) were abundant in both the human SAN and RA, but overall more highly expressed in the RA than the SAN, which may be partially explained by the different functions of SAN vs RA tissue. These data are supported by a recent study by Petkova et al.^11^ showing that miR-1 and miR-133, along with 64 other miRs, were different in the SAN vs RA of six human cadaver hearts by qPCR, suggesting that the SAN has a unique miR expression pattern related to its pacemaker function. Moreover, we observed both miR-1 and miR-133 transcription levels were higher in the HF SAN than nHF by NGS. It is known that miR-1 and miR-133 can target multiple transcription factors involved in cardiac hypertrophy, fibrosis, and cardiomyocyte apoptosis^29^. IPA analysis showed that these miRs are predicted to target HCN1, HCN2, SCN2B, and Na^+^-Ca^2+^ exchanger. As such, their upregulation during HF may be associated with depression of SAN automaticity and conduction.

MiRs can be expressed with tissue specificity and are altered in response to various physiological conditions^15^. During HF, a miR could be altered differently in different tissues or cell types. For example, Goren et al. ^30^ found that miR-423-5p was elevated in the blood of chronic HF patients, and suggested that it could be used as a valuable biomarker for HF. In the current study we found lower expression of this miR in HF SAN but not in RA, suggesting a tissue specific change of the SAN miR profile. Furthermore, miR-1247-5p was downregulated by HF in both SAN and adjacent RA detected by NGS. A recent study by Tran et al.^31^ showed that elevated circulating level of miR-1247-5p was associated with both echocardiographic markers of cardiac remodeling and prevalent HF and that it can potentially mediate cardiac dysfunctions in part through the p53 and TGF-β signaling pathways. Importantly, we observed a diminished difference of miR and mRNA profiles between SAN and RA in HF, which may result not only in suppressed SAN function but in increased ectopic automaticity of the RA. However, this suggestion requires further investigation. NGS analysis showed that miR-204-5p is higher in SAN than RA in both nHF and HF groups, which highlights its potential use as a tissue biomarker for distinguishing SAN and RA miR profiles in both nHF and HF conditions. miR-204 has been reported to play an important role in many cardiovascular diseases, such as hypertrophic cardiomyopathy^32^, myocardial ischemia–reperfusion injury, cardiac apoptosis and autophagy^33^. However, its effect on SAN function is still unknown and needs further study. Furthermore, revealing the functional relevance of the significantly altered miRs studied herein during pathological conditions may provide novel insights into crucial gene regulation in HF, and help to develop therapeutic approaches to preserve SAN function in HF progression.

Since miRs could regulate gene expression by translational repression^14^, it is not a surprise that not all the miRs predicted mRNA targets were altered in opposite directions as these miRs (Online Table 2). The predicted targets may not necessarily be altered at the transcriptional level. In addition to matching significantly altered miR and mRNA data sets for pathway analysis, we further screened all altered miRs in HF SAN within IPA database to explore all potential targets affecting ion channels, ion-exchangers, connexins, and neurotransmitter receptors. We found that at least 27 miRs were predicted to target ion channels or connexins involved in SAN automaticity and conduction. As shown in Fig. 8, HF altered miRs are predicted to have multiple mRNA targets involved in SAN function. Among those miRs miR-486-3p has the most predicted targets, and it has been recently confirmed that miR-486-3p^11^ could downregulate HCN4 and depress SAN automaticity in animal models. In the present study, NGS showed that miR-486-3p is more highly expressed in the human RA than in the SAN and upregulated in the HF SAN. Similarly, Petkova et al. ^11^ also reported that miR-486-3p is more abundant in the RA vs SAN by qPCR in six human cadaver hearts. Further studies are needed to validate whether miR-486-3p can depress SAN automaticity and conduction in HF through other ion channels predicted by IPA.

Recent animal studies have investigated the therapeutic potential of anti-miRs targeting the mRNA of pacemaker channels altered during diseased conditions. For example, D’Souza et al.^9^ showed that selective knockdown of miR-423-5p by anti-miR could reverse exercise training induced HCN4 channel remodeling and sinus bradycardia in a mouse model. Similarly, Yanni et al.^12^ demonstrated that local delivery of anti-miR-370-3p restored both HCN4 mRNA and protein, and thus I_f_ current in the SAN pacemaker myocytes in a HF mouse model. Their study^12^ also showed that in-vivo silencing of miR-370-3p could blunt SAN bradycardia and partially restore ventricular function and reduce mortality in this mouse model. Overall, these studies emphasize the increasing possibility of using miR-based therapies to alter gene expression in diseased organs. However, experimental observations from animal SAN studies requires further validation in the human heart. Thus, revealing the specific miR profile of the human SAN and pathological changes during HF may help to define novel human SAN-specific therapeutic targets and to develop gene regulation approaches for HF treatment.

In the current study, we chose SCN8A (encodes Nav1.6) and related miRs to further validate the predicted miR-mRNA interactions. Our recent ex-vivo human study^14^ revealed a new SAN-specific therapeutic targets to be SCN8A (encodes the protein Nav1.6), which is downregulated during HF in human SAN. Moreover, our near-infrared optical mapping study proved that activation of Nav1.6 is an important backup mechanism for maintenance of SAN automaticity and conduction during stress^19^. In fact, Nav1.6 blockade caused prolongation of SAN cycle length and increased both SAN recovery time and conduction time, ultimately causing SAN exit block. Importantly, multiple miRs (e.g. let-7g-3p, miR-3200-3p, miR-486-3p) upregulated in human SAN during HF are predicted to decrease SCN8A (Nav1.6) expression (Fig. 8), which was further confirmed by our Luciferase reporter assay experiments (Fig. S4). Moreover, Luciferase reporter assays also showed a difference in inhibition efficiency, suggesting that one miR may be more dominant for the regulation of a particular gene, even when that same gene is regulated by multiple miRs. Based on the results described above, we suggest that inhibition of those upregulated miRs (e.g. let-7g-3p) inhibiting SCN8A in HF SAN could be potential targets for SND treatment through upregulation/preservation of functional Nav1.6 during HF. Future studies are required to validate the effects of those miRs on Nav1.6 expression and SAN function in experimental settings.

Recent work has emphasized the importance of GIRK1 and GIRK4 channels (encoded by KCNJ3 and KCNJ5, respectively), which are the downstream functional units of acetylcholine and adenosine signaling, to SAN function^34^. In addition, our previous study also revealed an adenosine-induced SND phenomenon in canine model^35^ and ex-vivo human hearts^17^ by near-infrared optical mapping. In the current study, NGS and PCR revealed downregulation of miR-1247-5p in the HF SAN, which is predicted to target KCNJ3 and KCNJ5. Furthermore, the miR-mRNA binding site of miR-1247-5p to KCNJ5 is confirmed by our Luciferase reporter assay experiments (Fig. S5). As such, mimics of miR-1247-5p may potentially be used to downregulate KCNJ channels, as alternatives to GIRK channels blockers (e.g. tertiapin^17^), and reverse the pathological response of the SAN to adenosine during HF^36,37^.

The background complexity of the HF pathophysiology may add to the variation in miR profiles. The pathophysiological mechanisms underlying SND during HF could be complex due to HF induced multiple molecular and structural remodeling, involving ion channels, neurotransmitter receptors, scaffolding proteins and cytoskeleton proteins. The present study is the first step to reveal the different transcriptomic profile (mRNA and miR) of human SAN in HF vs nHF hearts, and provide potential molecular targets for regulation of SAN function in HF patients. Moreover, in the current study, 80% of the HF group samples are male, due to the availability of transplanted hearts with intact SAN pacemaker complex (74% transplanted human hearts obtained from The Ohio State University Cardiac Transplant Team in the last several years were male^20^). The effects of sex on SAN miR and mRNA profiles need to be further studied in a larger sample with balanced male and female samples.

The complexity of miR-mRNA gene regulation networks brings a major challenge for the selection of proper therapeutic targets and avoidance of off target effects. For example, as shown in our study and by other groups^38^, one miR could target multiple mRNAs and/ one mRNA could be regulated by multiple miRs. In future studies, in-vivo and ex-vivo animal and human studies should be conducted to validate functional effects of identified miRs and develop optimized local delivery methods to regulate target genes within specific SAN pacemaker compartments.

## Conclusions

Our study reveals comprehensive mRNA and miR profiles and associated signaling pathways in nHF and HF human SAN pacemaker tissue. The miR and mRNA profiles of both the SAN and RA were significantly altered in HF hearts. At least 27 miRs were found to be altered in the HF SAN and are predicted to regulate major ion channels and receptors responsible for human SAN automaticity and conduction. Our results suggest that miR-mRNA alterations in the human HF SAN could lead to tachy-brady arrhythmias and provide novel SAN-specific therapeutic molecular targets to restore impaired SAN pacemaking and conduction.

## Methods

An expanded Methods section can be found in the Supplemental Information file.

### Human tissue collection

Human hearts from both female and male, adult transplant patients (HF, n = 10, 39-64y.o.) with implantable pacemakers/ICD, and nHF human donor hearts (nHF, n = 9, 20-68y.o.) were obtained from The Ohio State University Cardiac Transplant Team and the LifeLine of Ohio Organ Procurement Organization in accordance with guidelines of The Ohio State University Institutional Review Board (IRB). All ex-vivo human heart tissue experiments were approved by The Ohio State University IRB and in compliance with all relevant ethical regulations. Informed consent for tissue collection was obtained from all transplant patients and/or their legal guardians and families of donors. No organs/tissues were procured from prisoners. For patient information, please see Table S1. Explanted human hearts were cross-clamped and stored in cold cardioplegic solution (4 °C) during transport and dissection. The whole SAN pacemaker-conduction complex, which included adjacent atrial tissue, was stored at − 80 °C until use.

### Human SAN pacemaker tissue isolation

The human SAN is a 3D intramural structure surrounded by atrial tissue^17,39–41^. However, there is a lack of SAN-specific markers for distinguishing the SAN from the atria at the transcriptional level. In our previous studies^17–19^, we developed a method to isolate pure SAN pacemaker tissue in order to define human SAN-specific protein and transcriptomic profiles, and to study HF associated profile changes.

As we described previously^18,19^, cryo-blocks of SAN tissues were cut into ~ 4–8 mm long blocks perpendicular to the epicardial surface (Fig. 1). Cryo-sections were collected from each end of the cryo-blocks at 14-16 µm thick. Masson’s trichrome staining and double immunolabeling with Connexin-43 (Cx43) and α-actinin were performed on cryo-sections and used to guide SAN tissue collection from each cryo-block. Sixteen-gauge (1.3 mm I.D.) biopsy needles were used to accurately collect SAN tissue along the SAN artery within the Cx43-negative area. RA myocardial tissue was collected from the same cryo-block.

### RNA and protein isolation

Total RNA was isolated from paired SAN and RA samples for HF (n = 10) and nHF (n = 9) hearts, as described previously^19^. After the SAN or RA samples were digested with cell lysis buffer, for each sample the upper aqueous layer was used for RNA isolation, and the lower organic layer was used for protein isolation. SAN samples with 260/280 > 1.8 and RIN > 5.8, and their paired RA samples were used for NGS.

### Next generation sequencing of miR and mRNA

SAN and paired atrial tissue of HF (n = 4) and nHF (n = 3) were used for next generation sequencing (NGS) through QIAGEN Genomic Services. For RNA quality control, the ratio of absorbance at 260 nm and 280 nm, and RNA Integrity Number (RIN) were measured by Nanodrop and TapeStation RNA Screen Tape (see Table S2). The detailed information for miR and mRNA library preparation and quality control are provided in Supplemental Information file and Fig. S6. For miR sequencing, a read count was generated for each miR annotated by miRbase_20^42^. For mRNA from RNAseq, gene expression was quantified for genes annotated by Homo_sapiens.GRCh37.75 from Ensembl using featureCounts^43^ of the subread package v1.5.1, which was set up to count the primary alignment for multimapped reads. For miR and mRNA comparisons, genes were filtered to retain those with at least 2 CPM (Counts per Million mapped reads) in half of the samples.

Quantitative PCR (qPCR) was used to validate miRs detected by NGS within SAN and RA samples of HF (n = 10) and nHF (n = 9) hearts. The primer assays for miRs (Thermo Fisher Scientific) are listed in Table S3.

### Bioinformatics analysis

The Ingenuity Pathway Analysis (IPA, Winter Release December 2019, https://analysis.ingenuity.com/pa/installer/select, Qiagen) was used to predict miR-mRNA targets or discover potential novel regulatory networks. Core analysis was performed with genes filtered for FDR < 0.05. miR Target filter analysis was used to identify mRNAs with altered expression targeted by miRNAs with altered expression. Specific attention was focused to miRs targeting mRNAs involved in human SAN automaticity and conduction. TargetScan predicted biological targets of miRs by searching for the presence of sites that match the seed region of each miR. PCA was used to explore sample clusters arising naturally based on the expression profile.

### Plasmids preparation and Luciferase reporter assay

A synthetic oligonucleotide containing the sequence of human SCN8A 3′-UTR (NCBI Reference Sequence: NM_014191.4) or human KCNJ5 3′-UTR (NCBI Reference Sequence: NM_000890.5) was assembled into the Firely/Renilla Dual-Luciferase pmirGLO vector (Promega, E133A).

HEK293 cells (ATCC, Cat No. CRL-1573) were co-transfected with 0.6 µg of pmirGLO-SCN8A (or KCNJ5) 3′UTR plasmid DNA and 3-6 pmol of the respective tested miRs or negative control miR per well of 24-well plate, using Lipofectamine 2000 (Invitrogen, Cat No. 11668027). Each transfection was performed in triplicate and repeated three times with an independent batch of cells. All reporter assays were done using the Promega Dual-Luciferase Assay System (Promega, Cat No. E2940) at 48 h post-transfection. Luminescence was analyzed based on the Firely/Renilla activity ratio.

### Statistical analysis

For NGS data, counts were normalized with voom, and then limma^44^ was used to perform differential expression analysis between groups of samples. A gene was considered significant when the FDR was less than 0.05. Plots were generated in R 3.6.3. qPCR data are presented as mean ± SD. Statistical analysis was performed with Graphpad 8.0. Shapiro–Wilk was used for normality test. One sample t test was used to check the significance of SAN/RA ratio in immunoblotting data and miR mimic/scramble ratio in Luciferase assay data. T-test (two tailed), and ANOVA were used to compare variations between two or multiple groups. If the data were not normally distributed, the Welch’s correction or the Mann–Whitney tests were used.

## Supplementary Information

Supplementary Information 1.

Supplementary Information 2.

Supplementary Information 3.

## Data Availability

The datasets generated during and/or analyzed during the current study are available from the corresponding author on reasonable request.
