# Supplementary material for: Altered microRNA and mRNA profiles during heart failure in the human sinoatrial node
Source: Sci Rep. 2021 Sep 29;11:19328. doi: 10.1038/s41598-021-98580-x (PMC8481550; doi:10.1038/s41598-021-98580-x)
Supplement: Supplementary file 1 — Supplementary Information 1. [file 41598_2021_98580_MOESM1_ESM.pdf]

Altered MicroRNA and mRNA Profiles during Heart Failure in the Human Sinoatrial Node

Li et al: Transcriptome in Failing Human SAN

Authors: Ning Li, MD, PhD,[1,2]; Esthela Artiga[1,2], MS; Anuradha Kalyanasundaram[1,2], PhD; Brian J. Hansen, PhD[1,2]; Amy Webb, PhD[3]; Maciej Pietrzak, PhD[3]; Brandon Biesiadecki, PhD [1,2], Bryan Whitson, MD, PhD[4]; Nahush A. Mokadam, MD[4]; Paul ML. Janssen, PhD[1]; John D. Hummel, MD[5]; Peter J. Mohler, PhD[1,2]; Halina Dobrzynski, PhD[6,7]; and Vadim V. Fedorov, PhD[1,2*]

Affiliations: 1 - Department of Physiology & Cell Biology, 2- Bob and Corrine Frick Center for Heart Failure and Arrhythmia, Dorothy M. Davis Heart & Lung Research Institute, 3 - Biomedical Informatics Shared Resources, 4 -Department of Surgery, Division of Cardiac Surgery, 5 - Department of Internal Medicine, Division of Cardiovascular Medicine; The Ohio State University College of Medicine and Wexner Medical Center, Columbus, OH, USA; 6 - Division of Cardiovascular Sciences, University of Manchester, Manchester, UK. 7- Department of Anatomy, Jagiellonian University Medical College, Cracow, Poland.

SUPPLEMENTAL INFORMATION

Supplemental Methods

Human tissue collection

Human hearts from both female and male, adult transplant patients (HF, n=10, 39-64y.o.) with implantable pacemakers/ICD, and non-failing human donor hearts (nHF, n=9, 20-68y.o.) were obtained from The Ohio State University Cardiac Transplant Team and the LifeLine of Ohio Organ Procurement Organization in accordance with guidelines of The Ohio State University Institutional Review Board (IRB). All ex-vivo human heart tissue experiments were approved by The Ohio State University IRB and in compliance with all relevant ethical regulations. Informed consent for tissue collection was obtained from all transplant patients and/or their legal guardians and families of donors. No organs/tissues were procured from prisoners. These organizations provide de-identified, coded human tissue. Therefore, investigators are unable to link any specimen to any protected health information or individual. **Table S1** shows available clinical history.

Explanted human hearts were arrested and cooled to 4°C in the operating room following cross-clamping of the aorta. Hearts were stored in cold cardioplegic solution (4°C) during transport (5-30 minutes) and dissection in the experimental lab. The whole SAN pacemaker complex region adjacent atrial tissue was pinned to silicone pads, embedded in frozen medium O.C.T. (Fisher Scientific), frozen and stored at -80°C until use.

Human SAN isolation

As we described previously[1, 2], frozen cryo-blocks of SAN tissues were cut into head, center, and tail blocks (each ~4-8 mm long) perpendicular to the epicardial surface (**Fig. 1**). 16G (1.3mm I.D.) biopsy needles were used to accurately collect SAN tissue along the SAN artery within the Cx43-negative area. About 10~15mg of pure SAN tissue could be collected from each cryo-block. Adjacent right atrial (RA) tissue was collected from the same SAN center cryo-block. Histology and immunostaining images were used to guide SAN tissue collection from each cryo-block. Cryo-sections were collected from each end of the SAN center cryo-blocks at 14-20μm thickness. Masson's trichrome staining and Connexin43 (Cx43)/α-actinin double immunostaining were performed on cryo-sections.

RNA and protein purification

Total RNA was isolated from paired SAN and RA samples using the mirVana miRNA Isolation Kit, with phenol (Life Technologies, Cat#-AM1560) according to the manufacturer's instructions. RNA purity and quantity was determined using a NanoDrop ND-2000 spectrophotometer (NanoDrop Technologies). Samples with an OD260/280 reading between 1.8 and 2.1 were used[2] for further experiments.

After the SAN or RA samples were digested with cell lysis buffer (following the protocol of RNA isolation), the organic layer of each sample was used for protein isolation. Total protein was precipitated with isopropanol and then washed twice with 0.3M Guanidine hydrochloride/95% ethanol. Final protein was precipitated with pure ethanol and re-suspended in 2x urea buffer (10µl buffer per 1mg tissue). Protein yield was quantified using RCDC protein assay (Bio-Rad).

Next generation sequencing of miRNA and mRNA

SAN and paired atrial tissue of HF (n=4) and non-HF (n=3) were used for Next generation sequencing (NGS) through QIAGEN Genomic Services. For RNA quality control, the ratio of absorbance (260/280), and RNA Integrity Number (RIN) were measured by Nanodrop and TapeStation RNA Screen Tape (see **Table S2**). All samples used for NGS had 260/280 >1.8 and RIN>5.8. QIAseq miRNA Library Prep kit (Qiagen) was used for microRNA (miR) library preparation on Illumina NextSeq platform. During the QIAseq miRNA Library Kit construction process, specially designed 3' and 5' adapters are ligated to mature miRNAs. The ligated miRNAs are then reverse transcribed to cDNA using a reverse transcription (RT) primer with a Unique Molecular Index (UMI). No libraries are prepared from adapter–dimers. Following cDNA cleanup, library amplification occurs with a universal forward primer and indexing reverse primers. Following a final library cleanup, 1µl of the miRNA Sequencing Library is used for quality control on an Agilent Bioanalyzer using a High Sensitivity DNA chip. A typical miRNA library is approximately 180bp as shown in **Fig S6**.

The first step of analysis for miR is the removal of library and sequencing adapters (referred to as trimming). Trimming of adapters created a distribution of sequences with different lengths. Average number of reads are over 12 million reads per sample. Number of sequencing cycles (read length): 76 nt. Single-end read (up to 46bp insert + 19bp 3' linker + 10 UMIs). Reads representing miRNAs have a length of ~18-22 nucleotides, longer sequences of other origin have a length of ~30-50 nucleotides (i.e. rRNA, tRNA, mRNA, and Y-RNA fragments). Following sequencing and trimming, reads were analyzed for the presence of UMIs. All reads containing identical insert sequence and UMI sequence (insert-UMI pair) were collapsed into a single read.

These reads were passed into the analysis pipeline. For miR sequencing, a read count was generated for each miR annotated by mirbase_20[3].

TruSeq® Stranded mRNA Sample preparation kit (Illumina inc) was used for mNRA library preparation. For mRNA selection: Oligo-dT beads capture polyA tails were used (No UMIs). The mRNA stranded libraries were pre-amplified with PCR and purified (AMPure XP). The libraries size distribution was validated and quality inspected on a Bioanalyzer 2100 or BioAnalyzer 4200 tapeStation (Agilent Technologies). High quality libraries are pooled based in equimolar concentrations based on the Bioanalyzer Smear Analysis tool (Agilent Technologies). The library pool(s) were quantified using qPCR and optimal concentration of the library pool used to generate the clusters on the surface of a flowcell before sequencing on a NextSeq500) instrument (75 cycles) according to the manufacturer instructions (Illumina Inc.). Following sequencing, intensity correction and base calling (into BCL files), FASTQ files are generated using the bcl2fastq software (Illumina Inc.). For mRNA from RNAseq, gene expression was quantified for genes annotated by Homo_sapiens.GRCh37.75 from Ensembl using feature Counts[4] of the subread package v1.5.1, which was set up to count the primary alignment for multimapped reads. On average 33.5 million reads were obtained for each sample and the average genome mapping rate was 78.1%. For mRNA and miR comparisons, genes were filtered to retain those with at least 2 CPM (Counts per Million mapped reads) in half of the samples.

PCR analysis

Quantitative PCR (qPCR) was used to validate miRs detected by NGS in HF (n=10) and nHF (n=9) hearts. All samples were sued to compare the differences between SAN (n=19) and paired RA (n=19). For miR detection, 10ng of total RNA from each sample was reverse-transcribed using the TaqMan microRNA Reverse Transcription Kit (Thermo Fisher Scientific, Cat No.: 4366596) with specific RT primer from each assay in a 15µl RT reaction. The primer assays were purchased from Thermo Fisher Scientific (**Table S3**). The RT cycling conditions were 16°C for 30 min, 42°C for 30 min and 85°C for 5 min. Product (1.3µl) from RT reaction was amplified using TaqMan Small RNA Assay and TaqMan Universal PCR Master Mix (Thermo Fisher Scientific, Cat#-4324018) in a 20 µl PCR reaction. Quantitative PCR was performed using applied biosystems (Thermo Fisher Scientific), and cycling conditions were 95°C for 10min, followed by 40 cycles of 95°C for 15s and 60°C for 1min. Based on NGS analysis, miR-19b-3p is the most stable miR across all group, so it was used as an endogenous reference in addition to RNU48.

All samples were run in duplicate. Average threshold cycle (Ct) values were obtained using QuantStudio Design & Analysis software (Thermo Fisher Scientific), and amplification curves were analyzed to check for experimental errors. Data were calculated as $2^{-\Delta Ct}$, where ΔCt is Ct (test) - Ct (reference).

Bioinformatics analysis

The Ingenuity Pathway Analysis (IPA, Qiagen, Winter Release December 2019, https://analysis.ingenuity.com/pa/installer/select) was used to predict miR-mRNA targets or discover potential novel regulatory networks. Core analysis was performed with genes filtered for FDR<0.05. miR Target filter analysis was used to identify mRNAs with altered expression targeted by miRNAs with altered expression. Specific attention was focused on miRs targeting mRNAs involved in human SAN automaticity and conduction. TargetScan predicted biological targets of miRs by searching for the presence of sites that match the seed region of each miR.

Principal component analysis (PCA) was used to explore sample clusters arising naturally based on the expression profile. The 500 genes that have the largest coefficient of variation based on abundance estimations have been included in the analysis. The largest component in the variation is plotted along the X-axis and the second largest is plotted on the Y-axis.

Immunostaining and Immunoblot analysis

Cryo-sections were fixed with 4% PFA/PBS for 10min before blocking. Sections were permeabilized with 0.1% Triton X-100 (Sigma Aldrich), blocked with 10% goat serum, and then incubated with the primary antibodies overnight at 4°C. The following day, sections were incubated in secondary antibodies for 90min at room temperature. Finally, the sections were mounted with ProLong® Gold Antifade Mountant with DAPI (Life Technologies). The primary and secondary antibodies include: anti-HCN4 (1: 200, Alomone), anti-Cx43 (1:400, Sigma-Aldrich), anti-α-actinin (1:200, Sigma-Aldrich), Alexa Fluor 488 and Alexa Fluor 568 (1:200, Life Technologies). Sections were imaged using either the Olympus FV1000 or FV3000 Filter confocal microscopes. For the immunobloting analysis, equal amounts (20μg/sample) of protein were separated by SDS-PAGE using 4-20% tris-glycine gels and then transferred to 0.45μm low fluorescence PVDF membrane by methods previously described[2]. After blocking, membranes were incubated with various primary antibodies overnight at 4°C. Anti-HCN4 (1: 200, Abcam), anti-Cx43 (1:8,000, Sigma-Aldrich), and anti-α-actinin (1:10,000, Sigma-Aldrich) were used to verify the quality and purity of SAN sample. Anti-GAPDH (1:20,000, Sigma-Aldrich) was used as

a loading control. Subsequently, 1:2,000 diluted fluorescent DyLight conjugated secondary antibodies (Jackson ImmunoResearch Laboratories) were applied to membranes for 1 hour at room temperature. The specific bands were detected on a Typhoon 9410 imager.

Plasmids preparation

A Firely/Renilla dual luciferase miR target expression plasmid, in which the SCN8A or KCNJ5 3′ UTR was integrated downstream of the firefly luciferase gene, were prepared for the luciferase report assay experiments. A synthetic oligonucleotide of 1763bp length fragment containing the sequence of human SCN8A 3'-UTR (3655-5418, NCBI Reference Sequence: NM_014191.4) was assembled into a pMK-RQ Kanamycin resistant vector by Invitrogen. SacI and XbaI sites were incorporated in the 5' and 3' ends of the synthetic fragment to facilitate cloning. In subsequent cloning steps, 2µg of plasmid was digested with 1µl SacI-HF (NEB, Cat No. R3156S) and 1µl XbaI (NEB, Cat No. R0145S). Similarly, 2µg of the pmiRGLO dual luciferase miR target expression vector (Promega, E133A) were also digested with the enzymes SacI-HF and XbaI. DNA purification of plasmid DNA after the restriction/digestion steps was performed by cutting bands of appropriate size from a 1% agarose gel under UV-light and then purifying the DNA from the agarose gels with QIAEX II gel extraction kit (Qiagen, Cat No. 20021) according to the manufacturer's instructions. For ligation, a total of 200ng of DNA including both the linearized pmirGLO vector and the insert were incubated in a molar ratio of 1:3 along with 1µl of 10X ligase buffer and 1µl T4 DNA ligase in a 20µL reaction at 25°C for 15mins. 50µL of 5-alpha E. coli competent cells (NEB, Cat No. C3019H,) were transformed with 5µl ligated of the plasmid, and spread on Ampicillin resistance LB-agar plates before incubation overnight at 37°C. Next, a single bacterial colony was incubated in 5ml LB medium supplemented with ampicillin (final concentration of 100 µg/ml) overnight at 37°C in an orbital shaker incubator (250 rpm). Plasmid DNA was purified using QIAprep Spin Miniprep Kit (Qiagen, Cat No. 27104) according to manufacturer's instructions. For analytical restriction digest, plasmid DNA was incubated with the restriction enzymes SacI-HF and XbaI to confirm the presence of the correct 3'UTR inserts in the pmirGLO vector. Similar protocol was used to create pmirGLO-KCNJ5 3'UTR (1-2380, NCBI Reference Sequence: NM_000890.5) plasmid.

Cell culture and Luciferase reporter assay

HEK293 cells were obtained from ATCC (Cat No. CRL-1573) and maintained with DMEM containing 5% FBS and 0.1% Penicillin/Streptomycin in a humidified incubator supplied with 5%

CO_2 at 37°C. 4.0x10^5 cells per well were plated in 24-well plates 24-48 hours prior to transfection. Lipofectamine 2000 (Cat No. 11668027; Invitrogen) was used for transfection according to the manufacturer's instructions. For reporter assays investigating miRs, HEK293 cells were co-transfected with 0.6µg of pmirGLO-SCN8A 3'UTR or pmirGLO-KCNJ5 3'UTR plasmid DNA and 3pmol-6pmol of the respective tested miRs (e.g. miR-3200-3p, miR-486-3p, miR-652-5p, let-7g-3p, and miR-1247-5p mimic) or negative control miR (scramble) per well. Each transfection was performed in triplicate and repeated at least three times with an independent batch of cells.

All reporter assays were done using the Promega Dual-Luciferase Assay System (Promega, E2940). Reagents were prepared and stored according to the manufacturer's instructions. In brief, 48 hours post-transfection, growth media was removed from the cells before the addition of Dual-Glo Luciferase Assay Reagent. For a 24-well plate, cells were lysed with 150µL of Dual-Glo Luciferase Assay Reagent at 25°C, and then transferred to a 96-well plate in duplicates. Firefly luminescence was measured 20-30 minutes after lysis. Next, 75µL of 25°C Dual-Glo Stop & Glo Reagent were added to each well, and the mixture was incubated for an additional 20-30 minutes before measuring the renilla luminescence. Luminescence was collected using a Perkin-Elmer EnSpire 96-well plate reader and data were analyzed based on the ratio of Firely/Renilla activity.

Supplemental Tables

Table S1. Explanted Human Heart information

Heart No.	Age	Gender	Clinical History	Cause of Death/ Transplant
nHF1	68	Female	No cardiac disease, alcohol consumption	CVA/Stroke
nHF2	51	Female	HTN, alcohol consumption	ICH/CVA
nHF3	46	Female	No cardiac disease, alcohol consumption	ICH/CVA & SAH
nHF4	67	Male	HTN, alcohol consumption	Blunt Injury/IPH
nHF5	23	Male	No cardiac disease, alcohol consumption	Cardiac arrest
nHF6	20	Male	No cardiac disease	Drug overdose/ Anoxia
nHF7*	40	Male	No cardiac disease	Drug overdose/ Cardiac arrest
nHF8*	56	Female	No cardiac disease	CVA/Stroke, ICH
nHF9*	40	Female	No cardiac disease	Anoxia
HF1*	61	Male	NICM HF	Transplant
HF2	47	Male	ICM HF	Transplant
HF3	50	Female	ICM HF	Transplant
HF4*	64	Male	ICM HF, SND, AF	CVA/Stroke
HF5*	53	Male	NICM, AF	Transplant
HF6	70	Male	ICM HF, SND	Transplant
HF7*	47	Male	NICM HF, AF	Transplant
HF8	53	Female	NICM HF, AF	Transplant
HF9	40	Male	NICM HF	Transplant
HF10	46	Male	NICM, HF, AF	Transplant

*Indicates hearts used for NGS analysis. Abbreviations: AF, atrial fibrillation; CVA, cerebrovascular accident; HF, heart failure; HTN, hypertension; ICH, intracerebral hemorrhage; ICM, ischemic cardiomyopathy; IPH, idiopathic pulmonary hemosiderosis; NICM, non-ischemic cardiomyopathy; PM, pacemaker; RA, right atrium; SAH, subarachnoid hemorrhage; SND, SAN dysfunction.

Table S2. Quality control of the RNA used for NGS

Sample ID	A260/A280	RIN
nHF7 RA	1.9	8.1
nHF7 SAN	2.0	7.3
nHF8 RA	1.8	8.6
nHF8 SAN	2.0	7.1
nHF9 RA	1.9	8.6
nHF9 SAN	1.9	7.7
HF1 RA	2.0	7.5
HF1 SAN	1.9	6.3
HF4 RA	2.0	7.8
HF4 SAN	2.0	6.4
HF5 RA	2.0	8.1
HF5 SAN	1.9	5.8
HF7 RA	1.9	7.1
HF7 SAN	2.0	6

Table S3. Primer assays used for qPCR

Assay name	Primer Assay ID
u48	001006
miR-19b-3p	000396
miR-204-5p	000508
miR-1-3p	002222
miR-133a-5p	464986_mat
miR-133a-3p	002246
miR-3200-3p	241643_mat
miR-1247-5p	002893
miR-486-3p	002093
let-7g-3p	002118
miR-652-5p	472084_mat

Online Table Legends

Online Table 1. List of all miRs and mRNAs in heat maps available as external data sets. miRs and mRNAs are listed in order corresponding to heat maps showed in Figures 2A, 2B, 3A, and 5A. Tables were generated with R 3.6.3.

Online Table 2. Significantly altered miRs and IPA predicted mRNAs altered in HF vs nHF human SAN available as external data sets. Table shows 7 miRs and 38 mRNAs which are both significantly altered in the HF human SAN. These 38 mRNAs have been experimentally confirmed to be targets of these 7 miRs according to the IPA database (Winter Release December 2019, Qiagen).

Online Table 3. miR abundance (top 50) in SAN and RA of HF and nHF human hearts available as external data sets. Relative miR expression levels in HF and nHF SAN and RA samples listed in order of abundance. miR abundance is presented as the log2 voom normalized counts as described in the methods.

Online Table 4. Ion channels and related pathways predicted to be targets of significantly altered miRs in HF vs nHF human SAN available as external data sets. List of all miRs targeting ion channels and connexins in the SAN along with their predicted mRNA targets and related pathways. mRNA targets were predicted by TargetScan human database. Table was generated with IPA (Winter Release December 2019, Qiagen).

Online Table 5. Significantly altered miRs presented in Figure 8 available as external data sets. List of all miRs with their seed sequence and experiment logFC. Table was generated with IPA (Winter Release December 2019, Qiagen).

Supplemental Figures and Legends

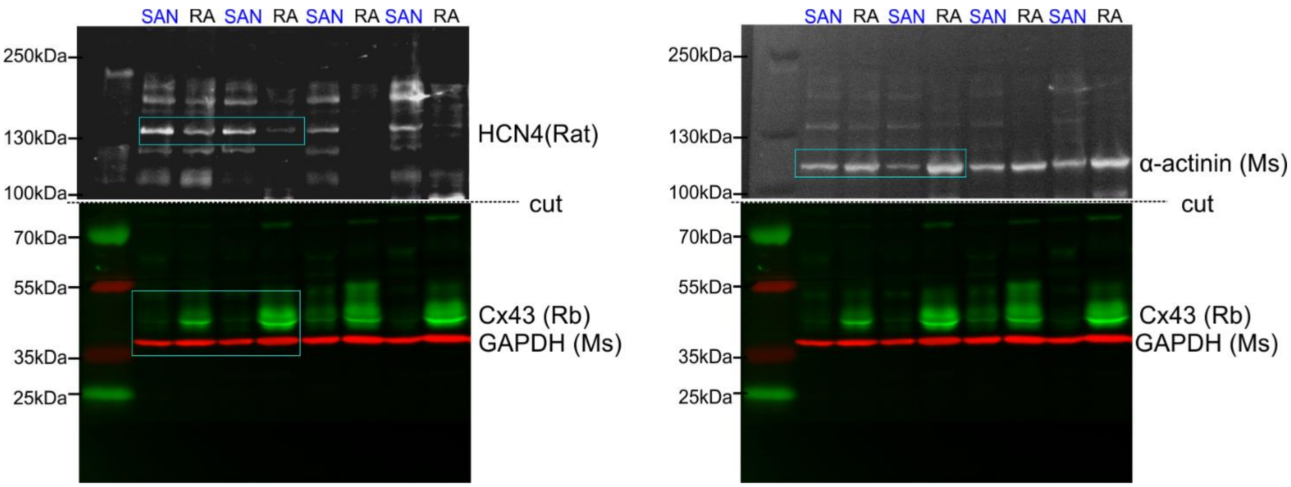

Fig S1. Full-length uncropped immunoblots for data presented in **Fig.1e**. Blue squares indicate the blots used in **Fig.1e.** Data are from the same blot which was cut at the dotted lines and processed with secondary antibodies detected either in the Red channel or Green channel, using the BioRad gel and blot imaging system.

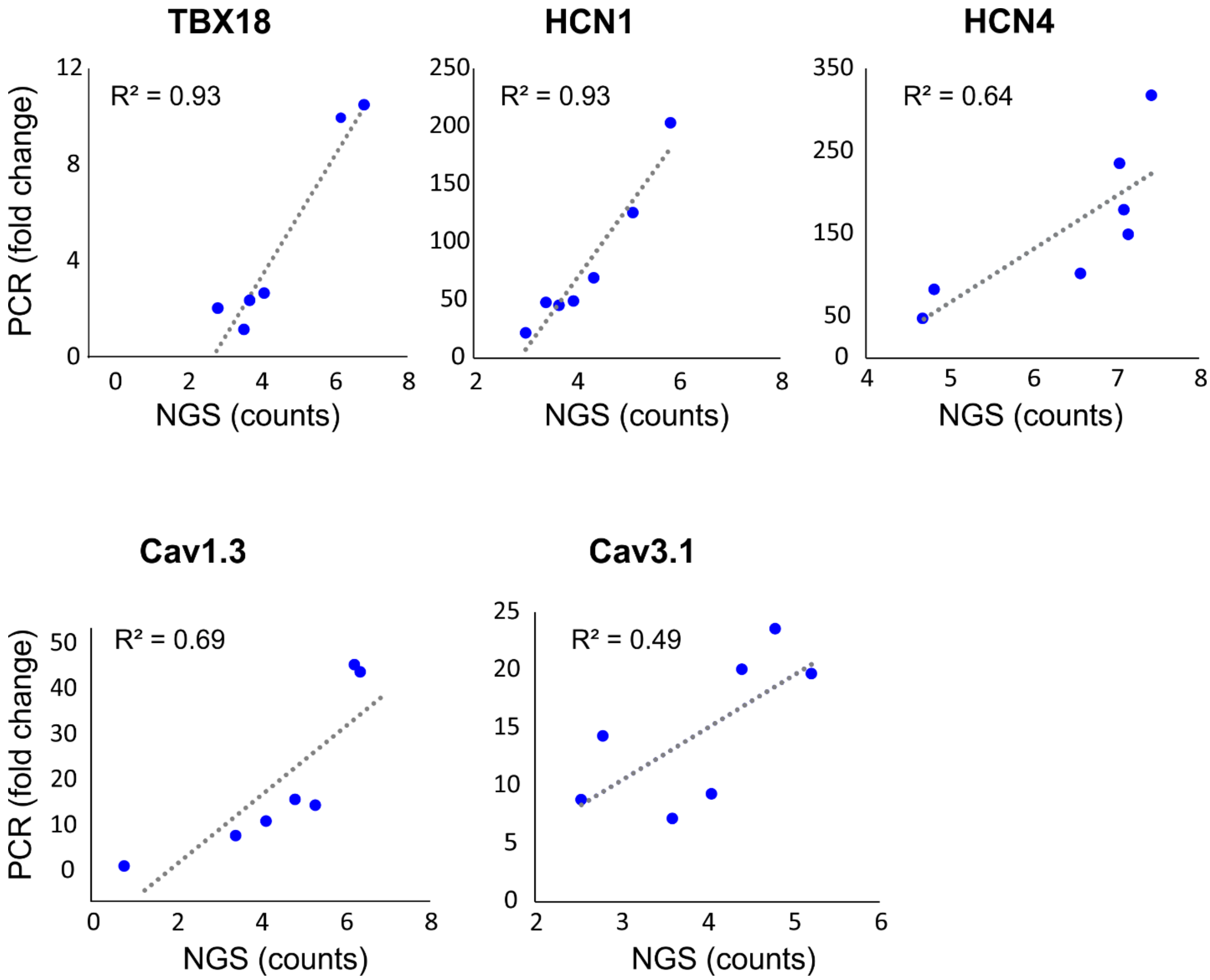

Fig S2. PCR and NGS correlation of key mRNAs. Correlation of PCR (fold change) and NGS (normalized gene counts) of some key mRNAs in SAN samples used for NGS (n=7).

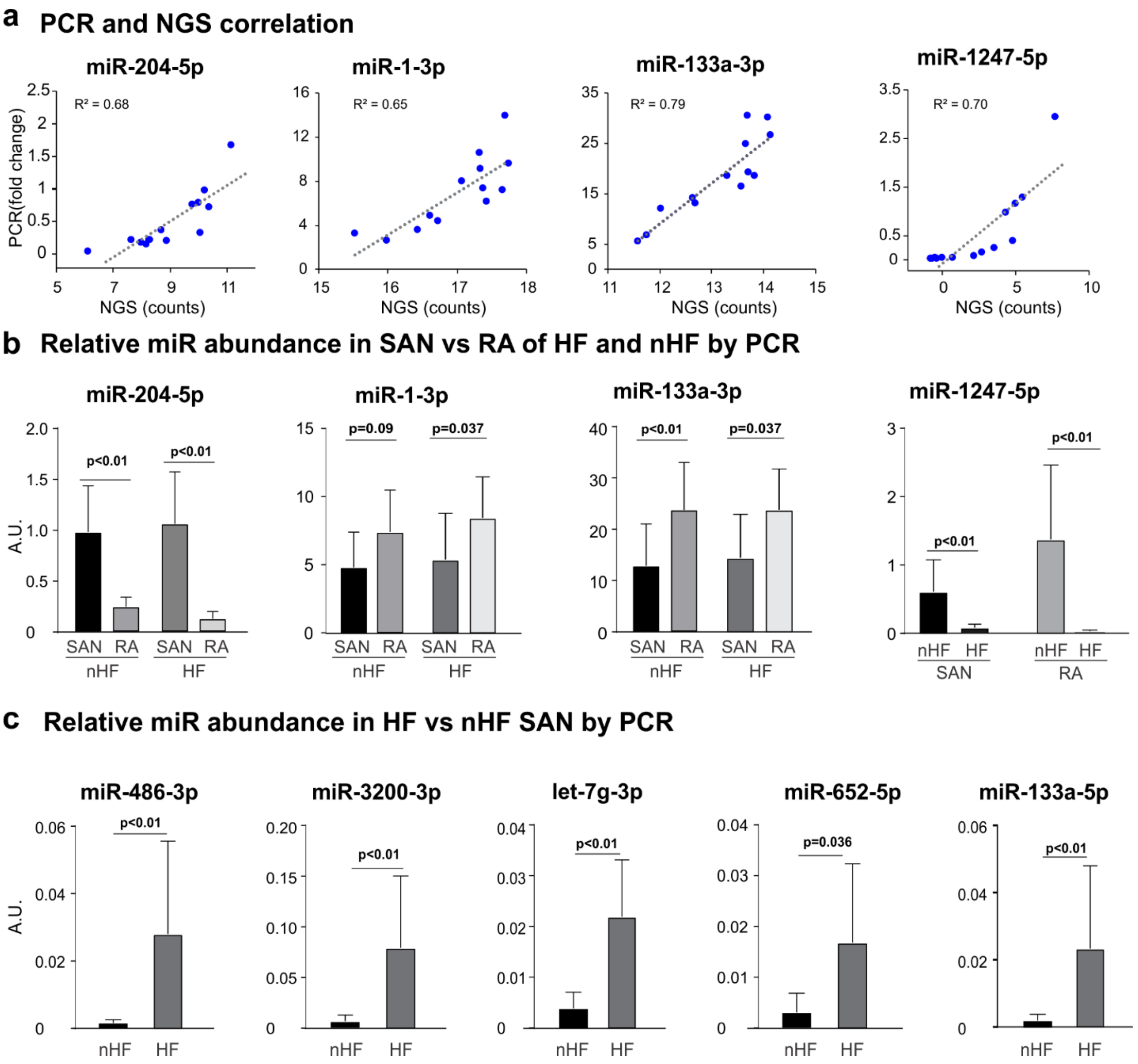

Fig S3. PCR analysis of altered miRs in nHF vs HF human SAN and RA. (a) Correlation of PCR (fold change) and NGS (normalized gene counts) of some key miRNAs in all SAN and RA samples used for NGS (n=14). qPCR results of selected miRs that are (**b**) differently distributed in human SAN and RA (n=19), and (**c**) significantly altered in the SAN of HF (n=10) vs nHF non-alcohol users(n=4) as detected by NGS and which are predicted by Ingenuity Pathway Analysis to target ion channels, receptors and connexins responsible for human SAN automaticity and conduction. qPCR data are presented as mean ± SD, and statistical analysis was performed with Graphpad 8.0. Shapiro–Wilk was used for normality test. Two groups were analyzed using Student's t-test (two tailed). If the data were not normally distributed, the Welch's correction (for SAN vs RA paired data) or Mann-Whitney test (for HF vs nHF non paired data) was used instead of t-test. HF, heart failure; nHF, non-heart failure; RA, right atrium; SAN, sinoatrial node.

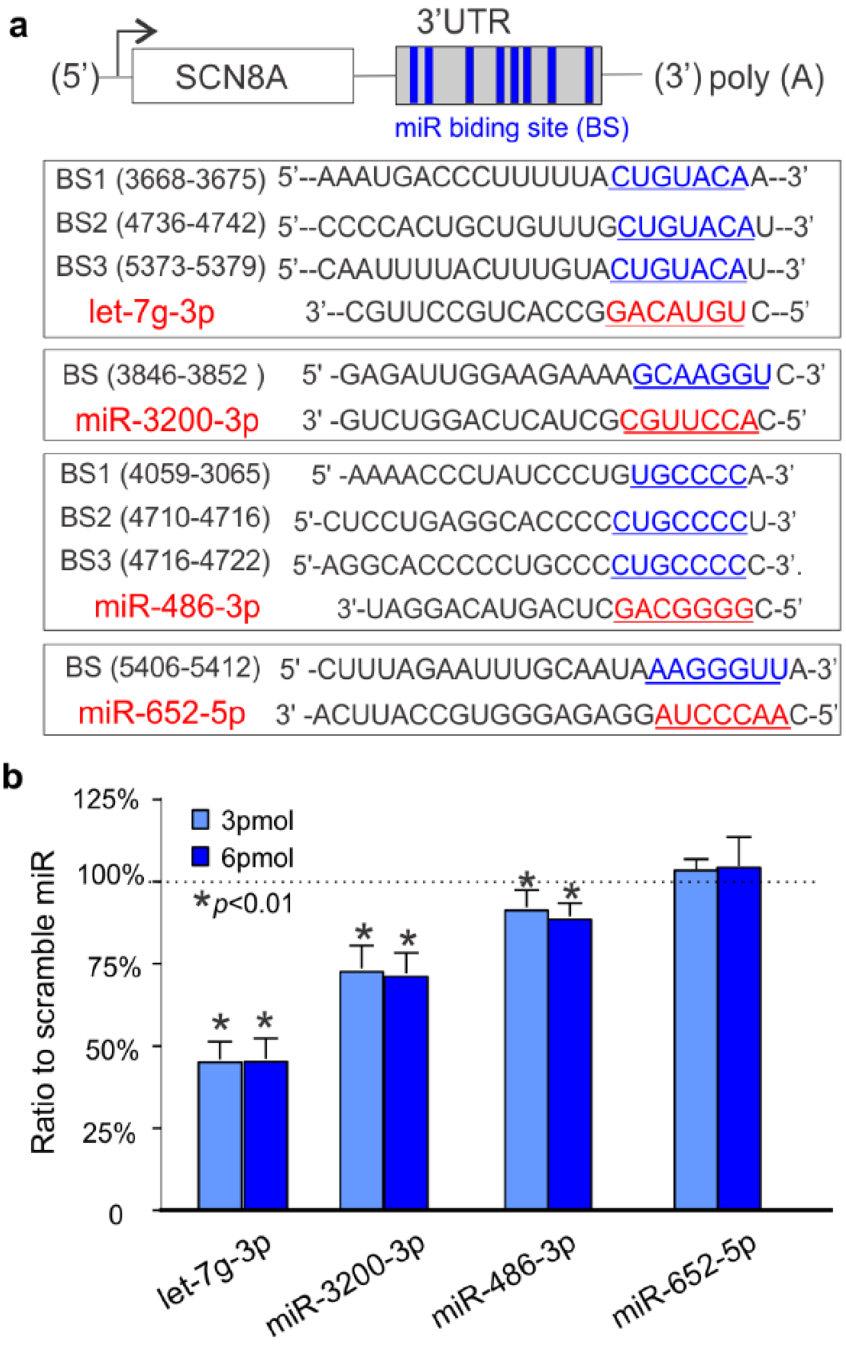

Fig S4. Validation of miRs targeting the neuronal sodium channel SCN8A involved in human SAN conduction. (**a**)Sequence of mature miR mimics and their binding sites at 3'-UTR of SCN8A. (**b**) Luciferase activity recorded 48h after transfection of HEK293 cells. n=9 for each miR. * indicates p<0.01 compare to scramble miR of the same concentration. Data are presented as mean ± SD, and statistical analysis was performed with Graphpad 8.0. Shapiro–Wilk was used for normality test. One sample t test was used to compare the ratio of each miR mimic to scramble miR. One-way ANOVA was used to compare the effects of different miRs.

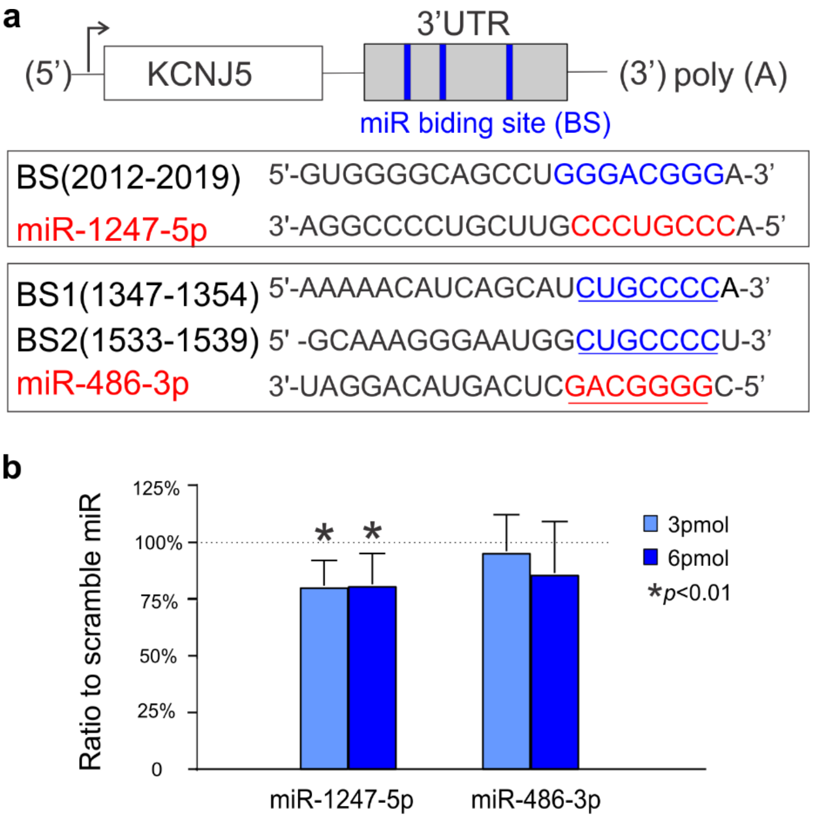

Fig S5. Validation of miRs targeting the KCNJ5 (GIRK4) involved in human SAN automaticity and conduction. (**a**) Sequence of mature miR mimics and their binding sites at 3'-UTR of KCNJ5. (**b**) Luciferase activity recorded 48h after transfection of HEK293 cells. n=11 for each miR. * indicates $p<0.01$ compare to scramble miR of the same concentration. Data are presented as mean ± SD, and statistical analysis was performed with Graphpad 8.0. Shapiro–Wilk was used for normality test. One sample t test was used to compare the ratio of each miR mimic to scramble miR.

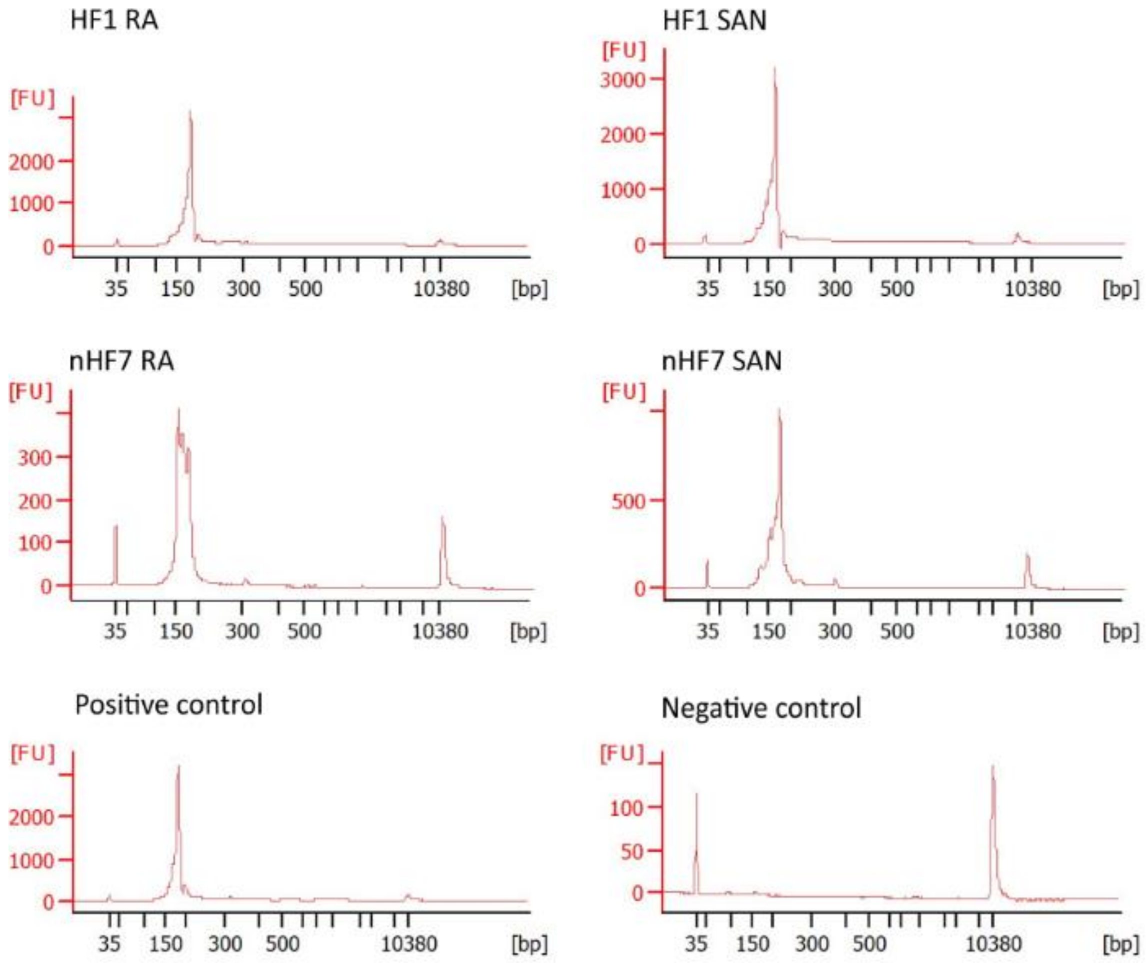

Fig S6. Electropherogram of miR library. Representative electropherograms showing miRNA library quality of SAN and RA samples in comparison to the positive and negative controls. HF indicates heart failure; nHF, non-heart failure; SAN, sinoatrial node.

Supplemental References

1. Li N, Csepe TA, Hansen BJ, Dobrzynski H, Higgins RS, Kilic A, Mohler PJ, Janssen PM, Rosen MR, Biesiadecki BJ, et al. Molecular Mapping of Sinoatrial Node HCN Channel Expression in the Human Heart. *Circ Arrhythm Electrophysiol*. 2015;8:1219-1227.

2. Li N, Kalyanasundaram A, Hansen BJ, Artiga EJ, Sharma R, Abudulwahed SH, Helfrich KM, Rozenberg G, Wu PJ, Zakharkin S, et al. Impaired neuronal sodium channels cause intranodal conduction failure and reentrant arrhythmias in human sinoatrial node. *Nat Commun*. 2020;11:512.

3. Kozomara A, Birgaoanu M and Griffiths-Jones S. miRBase: from microRNA sequences to function. *Nucleic Acids Res*. 2019;47:D155-D162.

4. Liao Y, Smyth GK and Shi W. featureCounts: an efficient general purpose program for assigning sequence reads to genomic features. *Bioinformatics*. 2014;30:923-30.
